# Ecological Status of Algeciras Bay, in a Highly Anthropised Area in South-West Europe, through Metal Assessment—Part II: Biotic Samples

**DOI:** 10.3390/toxics12030166

**Published:** 2024-02-21

**Authors:** María José Casanueva-Marenco, María Dolores Galindo-Riaño, María Dolores Granado-Castro, Margarita Díaz-de-Alba

**Affiliations:** Department of Analytical Chemistry, Institute of Biomolecules (INBIO), Faculty of Sciences, International Campus of Excellence of the Sea (CEI-MAR), University of Cadiz, Campus Rio San Pedro, 11510 Puerto Real, Cadiz, Spain; mariajose.casanueva@uca.es (M.J.C.-M.); dolores.granado@uca.es (M.D.G.-C.); margarita.diaz@uca.es (M.D.-d.-A.)

**Keywords:** metal bioaccumulation, fish, metal pollution, pollution indexes, water, sediment

## Abstract

Biotic samples from Algeciras Bay (South-west Europe) were studied to assess its ecological status, complementing the previous abiotic monitoring of trace metals in water and sediments. This bay is a densely populated area with intense port traffic and is highly industrialised with metal inputs. To study the impact of this, Zn, Cd, Pb, and Cu contents were determined in tissues of benthic (*Solea senegalensis*) and benthopelagic species (*Scorpaena porcus*, *Trigloporus lastoviza,* and *Diplodus sargus sargus*). Notable levels of Zn and Cu were found in the liver and gills of all fish species. Compared to international muscle guidelines, Pb sometimes exceeded the most restrictive values, outstanding *S. porcus* with 27% of samples above the permissible value. Metal pollution indexes revealed that the liver and gills of benthic species were more affected by metal pollution than benthopelagic species, especially in most industrialised sites. Particularly, *S. senegalensis* presented a higher accumulation factor from sediment of Cd and Cu in the liver (30.1 and 345.1), probably due to the close interaction as benthic species. Among the species studied, *S. senegalensis* and *D. sargus sargus* proved to be the best representative and useful bioindicators of metal-polluted environments as this bay. The results were consistent with the findings from the abiotic samples.

## 1. Introduction

Food safety is an issue of global concern, which makes it essential to determine the toxic elements in foods and their toxicological effects on human health. Therefore, organisations such as the World Health Organization (WHO), Food and Agriculture Organization (FAO), Environmental Protection Agency (EPA), and European Commission (EC) set maximum acceptable levels on foods in order to protect the health of consumers and promote good practices in the food trade [1,2].

The Mediterranean diet, which is highly valued internationally, is characterised by high fish consumption since it has been proven that it can reduce the appearance of cardiovascular and chronic inflammatory diseases, as well as certain types of cancer. Apart from the Mediterranean diet, local fish species are beneficial to socio-cultural aspects and the local economy since small-scale fisheries play an important role in the region [3,4].

Fish have high nutritional value, making them an important source of amino acids, proteins, and other essential elements for humans [3]. This concurs with the fact that fish can be affected by contaminants such as heavy metals, especially in aquatic environments influenced by anthropogenic activities. In general, aquatic organisms can accumulate sublethal metal concentrations that can cause damage to their biochemical, physiological, and reproductive functions, affecting long-term population survival [5,6]. Fish, being higher on the food chain, serve as potential bioindicators for aquatic environments [7,8,9,10], accumulating metals from food, water, and sediments [11]. Metal contents in different fish tissues can differ significantly due to metabolic mechanisms. Metal bioassimilation also depends on uptake pathways, tissue function, physiological exposure, reproductive activity, sex, and life stage [12,13,14]. Edible tissues are more critical to human health than other organs due to the biomagnification process throughout the food chain [15,16]. While muscle tissue is not highly active in accumulation, assessing its content is crucial since it is the main part consumed by humans [17].

Fish can be classified depending on where they live. Pelagic fish live in surface waters (up to 200 m), and demersal fish can be benthic if they live permanently on or near the seabed (irrespective of the depth of the sea) or benthopelagic if they inhabit close to the bottom (preferably in deep water) [18]. In general, benthic fauna has higher metal concentrations due to its direct contact with contaminant-rich bottom sediments together with their different habitats, feeding, and interaction among trace metals and species. The less spatial movement of benthic species can be a problem in contaminated areas [19]. It has been reported that benthic fauna, which can consume crustaceans, molluscs, and shrimps, has significantly higher levels of Cd, Cu, and Zn than pelagic. The presence of Cd is likely due to crustaceans’ tendancy to accumulate this element in high levels [20,21].

The biomonitoring studies in Algeciras Bay proposed in this work focused on the metals zinc (Zn), cadmium (Cd), lead (Pb), and copper (Cu), which are of great interest due to the anthropogenic activities affecting the bay, posing threats to aquatic life and human health. Industries, settlements, and ports in this area contribute to elevated metal levels. Zn sources include fossil fuel burning, traffic emissions (gasoline), and industrial/domestic wastewater. Cd sources may be metal smelting and refining, fuel burning, metal processing, and wastewater treatment. Pb presence is linked to petrochemical industries, coal combustion, traffic emissions (maritime transport), and marine engineering. Cu may result from traffic emissions (diesel oil), marine engineering, and industrial/domestic wastewater. Phosphate fertilisers may contribute to high Cd and Cu levels [22,23]. Pb and Cd, non-essential metals, are EU-WFD priority hazardous substances; Zn and Cu are essential and act as important co-factors in many biochemical processes but become toxic above a threshold concentration [24,25].

The aims proposed in this work were to determine the ecological status of Algeciras Bay using the following approach: (i) to determine the Zn, Cd, Pb, and Cu levels in gill, liver, and muscle tissues of fish species common in Algeciras Bay (benthic (*Solea senegalensis*) and benthopelagic species (*Scorpaena porcus*, *Trigloporus lastoviza* (accepted as *Chelidonichthys lastoviza*) and *Diplodus sargus sargus*)), studying the possible spatial and seasonal influences; (ii) to assess the potential health risks associated with the consumption of these metals by comparing the metal levels in the tissues with guideline and permissible levels; (iii) to compare the metal levels in fish with those from other ecosystems in the literature; (iv) to evaluate metal pollution indexes (MPI) to provide overall metal content for the different species and tissues at each site, and compare it with values from other ecosystems; (v) to evaluate the potential impact of the abiotic phases (liquid or solid) on metal accumulation in fish habitats, calculating the biota-water and biota-sediment accumulation factors (BAFs) and analysing correlations among fractions found in the aquatic compartments of the bay; for this last purpose, the studies of water and sediment from the bay, reported in *Ecological Status of Algeciras Bay, in a Highly Anthropised Area in South-West Europe, through Metal Assessment—Part I: Abiotic Samples*, were used.

## 2. Materials and Methods

### 2.1. Description of the Area and Sampling Sites

The Bay of Algeciras is an important industrialised area located on the Mediterranean coast of southwestern Spain, limited by Punta del Carnero (Algeciras) and Punta Europa (Gibraltar) [26]. This bay covers an area of about 9 km × 11 km^2^, with a maximum depth of almost 400 m [27]. Five cities with more than 275,000 inhabitants are located around the bay (Algeciras: 122,368, Los Barrios: 24,069, La Línea de la Concepción: 63,271, San Roque: 33,018) [28]; Gibraltar: 32,714; total: 275,440 [29]). The bay holds two important ports sited on Algeciras and Gibraltar, with intense marine traffic that can cause discharges and accidental spills [30], and also numerous industrial plants distributed along its coastline [27], including stainless steel manufacturing plants, refineries, and petrochemical installations, thermal power plants, ironworks, shipyards, and docks [26,31,32]. Furthermore, urban wastewater discharges may occur due to the high population density of the bay, coming from the main population centres of the cities of Algeciras, Los Barrios, San Roque, La Línea de la Concepción, and Gibraltar. The bay also receives the water discharge from the Guadarranque and Palmones rivers. The water of the bay has a high turnover because of its proximity to the Strait of Gibraltar, where the Mediterranean Sea and the Atlantic Ocean meet with strong currents. These geographical conditions could disperse pollutants into the water [26]. Nevertheless, marine pollution is a realistic risk and a major problem in this area subject to persistent anthropogenic pollution. The Bay of Algeciras exhibits high biodiversity, hosting approximately 50 fish species. Of these, only 35% are pelagic, while the remaining 65% consist of demersal fish (25% benthic and 40% benthopelagic), such as the species studied in this work [33].

**Figure 1:**
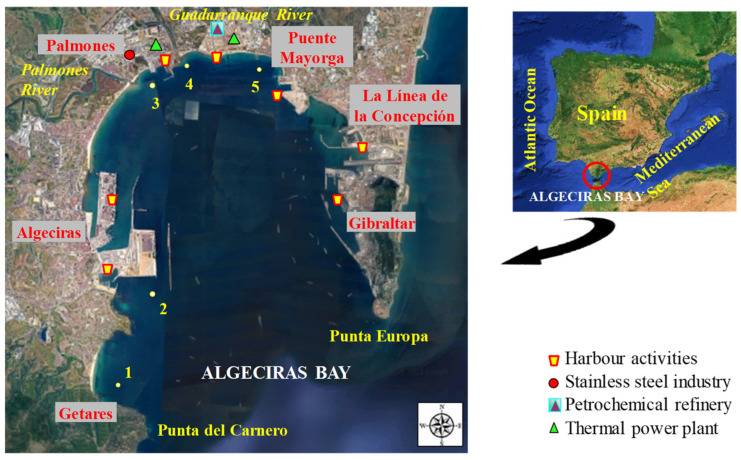
Map of Algeciras Bay showing the sampling sites and principal anthropogenic activities in the area.

Biotic samples (fish) were collected from 5 representative sampling sites (Figure 1): 1—*Getares beach* (control site with maritime traffic and limited urban influence), and four other pollution hotspots named 2—*Isla Verde* (with road and maritime traffic due to the port activity of the Port of Algeciras), 3—*Palmones* (area characterised by the presence of a steel manufacturing plant, a thermal power plant, the Palmones river, and urban influence), 4—*Guadarranque* (close to a Chemical Pole with refineries and a thermal power plant, apart from the presence of the Guadarranque river and urban influence), and 5—*Puente Mayorga* (close to power thermal plants, port activities, and maritime traffic from the Port of Gibraltar). The selection of these sites was based on previous studies [34], where the metal content in sediment samples from 17 sites along Algeciras Bay was studied. The different samples were consecutively collected during four samplings, as follows: sampling 1 (1st autumn), sampling 2 (1st spring), sampling 3 (2nd autumn), and sampling 4 (2nd spring). More information about sampling can be found in Table S1 of the Supplementary Material.

### 2.2. Equipment and Reagents

All analytical instruments and equipment used in this work are listed in Table S2 of the Supplementary Material.

All chemicals and standard solutions used for trace metal analyses were of Suprapur and Pro Analysis quality purchased from Merck (Darmstadt, Germany) or Sigma-Aldrich (Steinhein, Germany). The standard solutions required for the calibration curves were prepared by diluting 1000 mg/L commercial standard solutions.

### 2.3. Collection, Pretreatment and Analysis of Biotic Samples

Fish species of sole (*Solea senegalensis*), scorpionfish (*Scorpaena porcus*), streaked gurnard (*Trigloporus lastoviza*) and white seabream (*Diplodus sargus sargus*) were collected at sampling sites. Trammel nets and bottom trawling were used at night to conduct fishing operations and to catch the demersal species. Captured live fish were handled with care and transported in aerated tanks to the port, ensuring a journey of no more than 30 min, where they were anaesthetised and dissected. The size and weight of the fish sampled were measured, and tissue subsamples (liver, gills, and muscle) were quickly extracted from the species and stored at −80 °C using liquid nitrogen during transport to the laboratory. Samples were lyophilised and acid-digested by microwave heating, as reported previously [35], and metal concentrations were analysed using ICP-MS.

The experiments described comply with the Guidelines of the European Union Council (86/609/EU) and the Spanish Government (RD 1201/2005) for the use of animals in research.

The limits of detection of the metal analysis in the different tissues are shown in Table S3 of the Supplementary Material.

### 2.4. Quality Control and Quality Assurance

All experimental procedures were carried out using latex gloves and a second pair of disposable PE gloves, which are free of trace metals and usually used in clean rooms. Plastic and glass labware were cleaned using a 2 mol/L nitric acid bath overnight, followed by rinsing with ultrapure water and air drying in a laminar flow hood. The materials were finally sealed in polyethylene bags until use. Each sample was prepared and processed in duplicate and analysed in three replicates (*n* = 3) to ensure the reliability of the methods and measurements. In all cases, blank samples were performed following the same protocols described for samples. Standard solutions for metal calibration curves were prepared in matrices similar to the samples. Standards and blanks were also run between every 10 sets of samples for quality control of the measurements. The average values of the relative standard deviations (%RSD)—obtained from the three replicates of standards and samples—were most often <10%. The limits of detection (LD) of the metal analysis were determined (defined as *3·s*/*m*, where *s* is the standard deviation of 10 blank measurements and *m* is the slope of the calibration curve [36]; Table S3 of the Supplementary Material). The following certified reference materials were analysed following the same procedures as for the samples obtaining successful recoveries rates (Table S4 of the Supplementary Material): biological materials NRCC DOLT-3 (dogfish liver, recoveries of 94.7–97.4%) and DORM-2 (dogfish muscle, recoveries of 92.9–105.5%), purchased from the National Research Council of Canada (NRCC, Ottawa, ON, Canada).

### 2.5. Statistical Software

Statistical analyses of the obtained data were performed using the STATISTICA 7 software package (STATSOFT 2004, Inc., Tulsa, OK, USA). First, Levene and Brown-Forsythe tests were used to measure the homogeneity of the data, and the normality of results was checked by the Shapiro-Wilk test (*n* < 30) or the Kolmogorov-Smirnov test (*n* > 30). Some data were neither homogeneous nor normally distributed even when they were mathematically transformed (log x, log (1 + x), 1/x, 1/(1 + x), x^2^). In these cases, a series of non-parametric tests were carried out. The evaluation of significant differences of analysed metal levels within samplings and sites for the different samples was estimated using the parametric one-way ANOVA or the non-parametric Kruskal-Wallis test and the multiple comparison tests. The Pearson matrix was used to determine the correlation between the concentrations of the pollutants in the different environmental compartments for homogeneous and normal data, while the Spearman’s Rank correlation was employed for non-homogeneous and non-normal distributions. The results of the testing were considered significant at *p* ≤ 0.05. The spatial distribution maps for the Metal Pollution Indexes in different tissues for benthic and benthopelagic fish at each sampling site have been generated using ArcGis Desktop 10.8.2 (Copyright © 2021, Esri, Madrid, Spain).

## 3. Results and Discussion

### 3.1. Metal Content in Fish Samples

**Figure 2:**
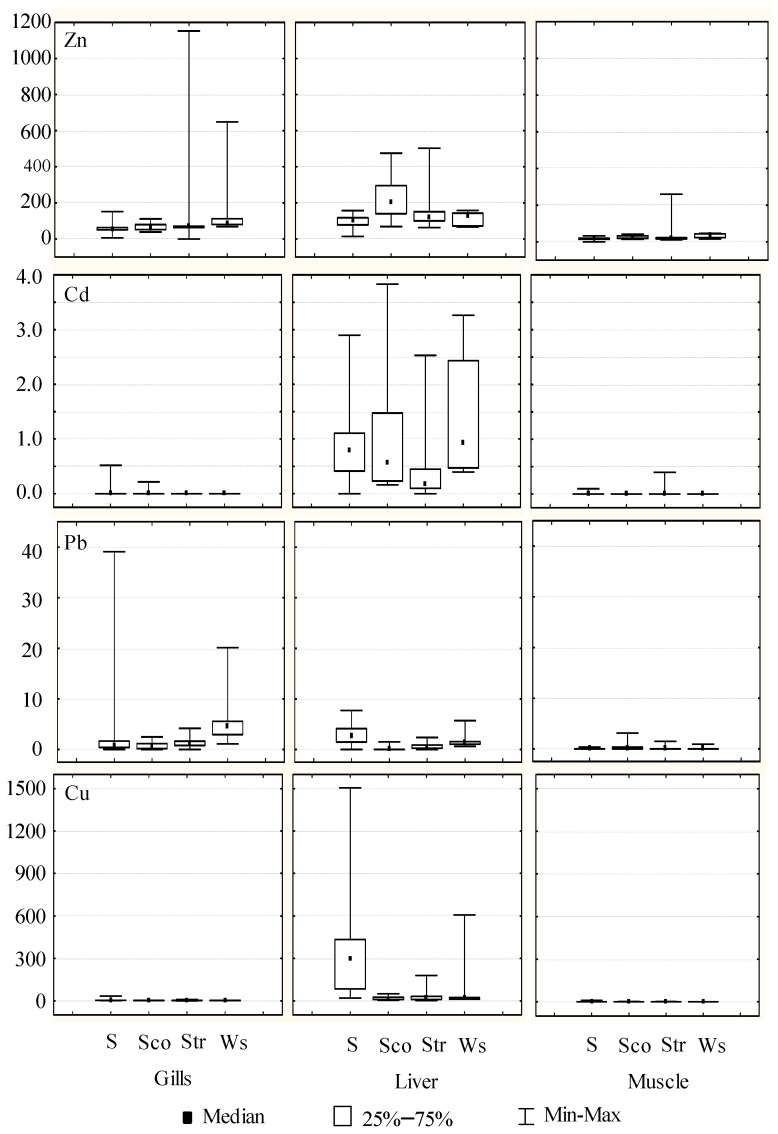
Box–Whisker plots for Zn, Cd, Pb, and Cu (mg/kg d.w.) in fish tissues (gill, liver, and muscle) taken from Algeciras Bay (S: sole; Sco: scorpionfish; Str: streaked gurnard; Ws: white seabream).

The total metal content in gills, liver, and muscle tissues was determined making use of different specimens of sole (*Solea senegalensis*) (*n* = 46), black scorpionfish (*Scorpaena porcus*) (*n* = 15), streaked gurnard (*Trigloporus lastoviza*) (*n* = 21) and white seabream (*Diplodus sargus sargus*) (*n* = 5). Table 1 shows the ranges of concentrations found as well as the average values in the different tissues at each sampling site (considering all species). For Zn and Cd, average metal concentrations were higher at sampling site 2 in liver tissues (206.8 and 1.36 mg/kg, respectively); for Pb were higher at sampling sites 1 and 5 in gills (3.05 and 2.58 mg/kg) and liver tissues (2.76 and 2.35 mg/kg); and these sampling sites also presented higher concentrations for Cu in the liver (313.3 and 268.4 mg/kg). On the other hand, Figure 2 depicts Box-Whisker plots for metal concentrations in the different tissues of each fish species using median values. In general terms, the highest concentrations were found in the following tissues and specimens: Zn in gills and liver of all species, Cd in liver of all species, Pb in gills and liver of sole and white seabream and Cu in liver of sole and white seabream. According to these experimental data, general metal content was classified as Zn ≥ Cu > Pb ≥ Cd. Regarding the metal accumulation in each tissue, it can be ordered as follows: Zn > Cu ≈ Pb > Cd (gills), Cu ≈ Zn > Pb ≈ Cd (liver) and Zn > Cu > Pb ≈ Cd (muscle). The higher levels of Zn and Cu compared to Cd and Pb can be explained by the fact that they are essential metals for fish, necessary for metabolic activities and normally easily absorbed by them [37]. The average weight and length of sampled fish were: (a) sole 197.1 ± 86.1 g and 27.0 ± 4.0 cm; (b) black scorpionfish 246.5 ± 121.8 g and 23.3 ± 5.5 cm; (c) streaked gurnard 268.9 ± 99.7 g and 30.5 ± 5.3 cm; and (d) white seabream 116.3 ± 40.9 g and 21.1 ± 9.4 cm. The relationship between metal concentrations and the length of fish was not found, probably due to the low variability of this morphological data in the samples (benthic length average: 27.0 ± 4.0 cm; benthopelagic length average: 26.6 0 ± 7.2 cm). The only exception was the Cd content in the liver of the benthopelagic species, which showed a negative correlation of −0.5344. This result for Cd has been described in the literature as likely due to size-specific metabolic rates associated with fish growth [38].

Liver and gill tissues are involved in fish xenobiotic transformation, storage, and elimination. For this reason, these organs are considered major targets for toxicity assessment. In contrast, muscle is an organ that does not tend to bioaccumulate this type of heavy metals as much and is less sensitive to detecting possible contamination of aquatic ecosystems [14,39,40].

Significant variations between sites and samplings for the different fish and tissues were observed using non-parametric Kruskal-Wallis ANOVA analysis: (a) for sites: Zn in gills of scorpionfish (sites 1–4, *p* = 0.02440 and sites 2–4, *p* = 0.02298), and Pb in gills of sole (sites 3–4, *p* = 0.02121); (b) for samplings: Cd in liver of streaked gurnard (samplings 1–3, *p* = 0.04271), Pb in gills of sole (samplings 3–4, *p* = 0.03715), Cu in gills of streaked gurnard (samplings 2–3, *p* = 0.04709), and Cu in liver of sole (samplings 1–4, *p* = 0.04159). The differences between sites were more significant than between samplings.

#### 3.1.1. Comparison with Guide Levels and Other Ecosystems

The results obtained for muscle samples were compared with limit values for muscle content found in the literature (by the Food and Agriculture Organisation of the United Nations (FAO), World Health Organisation (WHO), Ministry of Agriculture, Forestry and Fisheries (MAFF), European Commission (EC), European Union (EU), and Turkish Food Codex (TFC)) (Table 2). The conversion of the limit values from wet to dry weight has been performed using a factor of 0.208 (considering 79% moisture content) [17]. This table also shows the concentration ranges and the percentage of samples that surpass the most restrictive value.

The results showed that only 5% of streaked gurnard muscle samples exceeded the most restrictive values of Zn and Cd (FAO values: 143 and 0.24 mg/kg d.w., respectively), while all species exceeded the most restrictive value of Pb (FAO value: 0.11 mg/kg d.w.) with percentages of 13%, 27%, 19%, and 20% of sole, black scorpionfish, streaked gurnard, and white seabream muscle samples, respectively. The maximum limit of Cu was not exceeded. There are no reference or limit values for other tissues and therefore they could not be compared. However, as observed throughout this study, the concentrations in gills and liver would be higher than in muscle. The results for all tissues have also been compared with other studies found in the literature using sole, black scorpionfish, or white seabream species. No studies have been found for the specie-streaked gurnard. Table 3 shows the average concentrations as well as the calculated ratio between the concentrations in Algeciras Bay and other sites, where the positive values in red mean the times that our results are higher compared to the others, and the negative green values mean the times they are lower. 

Values from this study below the detection limit could not be compared. In general, the values in sole were not too different among them (up to 2.0 times higher and 2.2 times lower), with the exception of Pb in liver tissues, where the values of Algeciras Bay (this study) were up to 34.3 and 20.9 times exceptionally higher than those of Cádiz Bay and the Senegalese coasts, respectively [48,49]. No great difference was observed with respect to the Huelva Estuary, an estuary with evidence of metallic contamination. The values in black scorpionfish from Algeciras (this study) were especially higher than the others found for Pb in muscle [50,51,52,53,54]. The values were also especially higher for Cu in muscle. Cu values from Algeciras were up to approximately 38 times higher in gills, 39.2 in liver, and 14 times in muscle than in those reported for the Black Sea [53]. The high Zn values in Algeciras also stand out compared to the Black Sea [52] and the Tuscany coast [51]. For white seabream, the highest ratios were also found in the case of Pb, up to approximately 13.4 times higher in Algeciras than in the Cassidaigne canyon (muscle) [54], 11 and 10 times in muscle and liver, respectively, and 7.2 times for Zn (muscle) in the Canary Islands [57]. On the contrary, the level of Pb in the muscle of Algeciras was 57 times lower than in the Seixal Bay [59] because this bay was affected by several effluents non treated and showed a biomagnification phenomenon for Pb. Additionally, 10.6 times for Pb in muscle of white seabream from Cádiz Bay were found [61].

Therefore, in general, it can be concluded that the concentrations of Pb in Algeciras Bay were notably higher than in the others for the three species, as well as Cu values in comparison to values reported in some ecosystems for black scorpionfish or Zn in muscle for black scorpionfish and white seabream. This fact revealed a possible phenomenon of pollution by the non-essential element Pb in this industrialised area.

#### 3.1.2. Assessment of Fish Quality Using Metal Pollution Index (MPI)

The metal pollution index (MPI) of each tissue in fish was calculated in order to estimate the total amount of metals in the different tissues using the following equation [62]:

(1) MPI=(M1·M2·M3·… Mn)1n

where *M_n_* is the concentration of metal *n* (mg/kg dry wt.) in each tissue.

**Figure 3:**
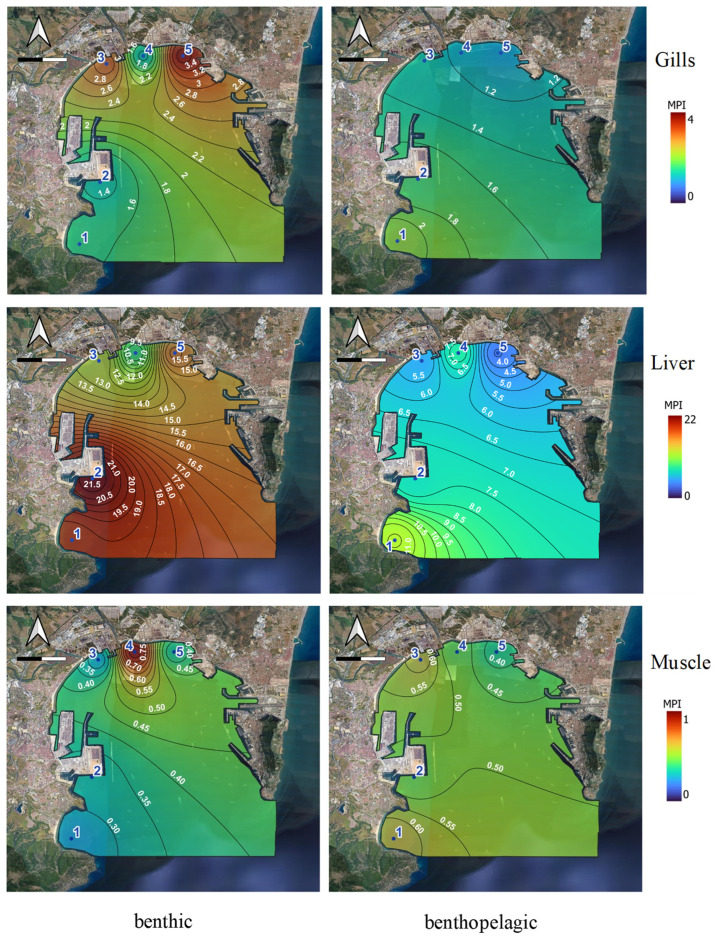
Metal pollution indexes for Zn, Cd, Pb, and Cu in tissues (gill, liver, and muscle) of the benthic and benthopelagic species at each sampling site.

The spatial distribution of the MPI index for the different tissues is represented for benthic and benthopelagic fish for the different tissues in Figure 3.

The lack of values in certain points is due to the fact that specific specimens of fish could not be caught. The MPI values can be ordered according to this trend: liver > gills > muscle, being more affected benthic than benthopelagic fish by metal contamination. For the liver, the high MPI values are similar throughout the bay, highlighting site 2 (Algeciras port). For the gills, higher values were obtained for sites 3 and 5, which were characterised by different industrial activities. These types of fish can be influenced by high concentrations of Cu and Pb in sediments (higher or very close to TEL levels, respectively). However, for muscle, there are no major differences due to the low variability of the index, which ranges between 0 and 1.

Also, several MPI indexes have been found or calculated (for the four metals under study) from the literature (Table 4) for sole (*S. senegalensis*) [35,48,49], black scorpionfish (*S. porcus*) [50,51,52,55], white seabream (*D. sargus sargus*) [58,59], Moroccan white seabream (*D. sargus cadenati*) [57], and other fish species [9,17,35,61,63,64,65,66,67].

The highest MPI values for gills in Algeciras corresponded to *D. sargus sargus* (3.71) and *S. senegalensis* (2.81). These metal pollution indexes were lower than those found for *S. senegalensis* (4.79) in Cadiz Bay (Spain) [48], *S. senegalensis* (6.86) and *S. aurata* (4.35) in the Huelva Estuary (Spain) [35] or *S. solea* (32.29) and *S. aurata* (22.81) in the Iskenderun Gulf (Turkey) [9], which showed high MPI values. For liver, the highest values in Algeciras corresponded to *S. senegalensis* (15.75) and *D. sargus sargus* (12.45), lower than those reported for *S. senegalensis* (17.85) and *S. aurata* (27.93) in the Huelva Estuary [35] or *S. solea* (80.87) and *S. aurata* (41.49) in the Iskenderun Gulf [9] with reported persistent contamination over time. For muscle, *D. sargus sargus* (3.41) and *S. porcus* (2.18) presented the highest values in Algeciras. They were higher than the rest found in the literature, except in comparison with the values in the Iskenderun Gulf (11.53 and 9.42) [9] or Seixal Bay (5.30) [59].

As previously stated, and based on these results, the liver and, secondly, the gills can be considered the main target tissues, compared to muscle, for the assessment of metal content in fish in most ecosystems. However, the values obtained for muscle samples in this study are high compared to the rest of the reported values. Among the studied fish species, *S. senegalensis* and *D. sargus sargus* could be considered representative and useful bioindicators of metal-polluted environments. The marine flatfish *Solea senegalensis* is one of the most abundant and representative species of the Atlantic coasts of Europe and Africa and is farmed in southern European countries, with high ecological and commercial values [68,69]. Thereby, it has been chosen for several environmental studies in order to assess environmental habitat quality [69,70,71,72,73,74,75,76,77,78,79,80,81].

### 3.2. Bioaccumulation Factors (BAFs)

The risks associated with metal levels in fish tissues were assessed by means of the bioaccumulation factor (BAF). This factor evaluates the bioaccumulation of a certain element with respect to the environmental matrices [82]. The biota-water accumulation factor (BWAF) can be calculated as the ratio between the concentration of metal ions in fish tissue (mg/kg, dry weight) and the concentration in water (µg/L), while the biota-sediment accumulation factor (BSAF) refers to the concentration in the sediment compartment (mg/kg, dry weight). It has been established that fish tend to accumulate metal content if BWAF > 1, but it is significant when it exceeds 100 or more [83]. On the other hand, tissues can be classified as macroconcentrators (BSAF > 2), microconcentrators (1 > BSAF < 2) or deconcentrators (BSAF < 1) [84].

The number and percentage of fish samples that surpassed the reference BWAF and BSAF values are indicated in Table 5. The most significant results are marked in red. In many cases, BWAF values were above 1, especially in the gills and liver of the species studied, and were above the value of 100 (BWAF > 100) for Zn in 83% of black scorpionfish liver samples as well as 55% of sole liver samples for Cu. These cases reflected an important bioaccumulation in the biota from water.

Regarding BSAF values, they were above 2 (BSAF > 2) in a high percentage of liver samples of sole (69% for Zn, 57% for Cd, and 92% for Cu), black scorpionfish (92% for Zn and 75% for Cd) and white seabream (100% for Zn and 67% for Cd), as well as in white seabream gills samples for Zn (80%). In these cases, these tissues can be considered as macroconcentrators of these metals. As microconcentrators (1 > BSAF < 2), the gills of sole (83%) and black scorpionfish (80%) and liver of streaked gurnard (65%) stand out for Zn. In general terms, the rest of the gills and muscle tissues revealed their deconcentrator nature (BSAF < 1).

The BWAF and BSAF values calculated for all samples can be found in Table S5 (Supplementary Material). In general terms, BWAF values were higher than BSAF values (except for Cd and Cu in the liver, where the opposite happens), and the liver presented the highest values compared to gills and muscle (the latter with the lowest values). Zn was the element with the highest values of BWAF and BSAF. Specifically, the maximum BWAF values obtained for gills were as follows: 1343.1 for Zn (an anomalous high value for site 1, white seabream), 1.7 for Cd (site 2, sole), 81.3 for Pb (site 1, white seabream), and 68.5 (mg/kg)/(µg/L) for Cu (site 5, sole); for liver: 586.2 for Zn (site 1, streaked gurnard), 19.3 for Cd (site 2, sole), 28.0 for Pb (site 1, sole) 319.3 for Cu (site 5, sole); and for muscle: 47.1 for Zn (site 2, white seabream), 4.1 for Cd (site 3, streaked gurnard), 11.4 for Pb (site 1, black scorpionfish), and 4.2 for Cu (site 5, sole). On the other hand, the maximum BSAF values for gills were as follows: 27.5 for Zn (site 1, streaked gurnard), 0.80 for Cd (site 2, black scorpionfish), 2.7 for Pb (site 1, sole), and 3.4 for Cu (site 5, sole); for liver: 12.0 for Zn (site 1, streaked gurnard), 30.1 for Cd (site 1, sole), 0.8 for Pb (site 5, sole), and 345.1 for Cu (site 1, sole); and for muscle: 3.7 for Zn (site 3, streaked gurnard), 2.2 for Cd (site 3, streaked gurnard), 0.14 for Pb (site 1, black scorpionfish), and 0.67 for Cu (site 3, streaked gurnard). It can be stated that fish, especially sole species, presented greater bioaccumulation of Cd and Cu from the sediment from where it seems that they have taken the most bioavailable fraction due to their benthic character.

Recent studies on bioaccumulation factors for different fish tissues have been found in the literature. Ahmed et al. studied bioaccumulation in edible tissues of seven important commercial species from the Meghna River Estuary in Noakhali district (southeastern Bangladesh), obtaining high mean BWAF values for Pb > Cr > As > Cd > Cu (mg/kg)/(mg/L), with a higher concentration of metals in demersal than in pelagic species [85]. Rubalingeswari et al. studied BWAF values in six species of commonly edible fish from the Adyar Estuary (India) and observed the highest BWAF values for Cu, Cr, and Zn, where Cu had a high biomagnification impact in all the fish studied, especially in the liver and muscle, while Zn showed greater biomagnification in the liver and gill tissues [86]. Kontas et al. investigated the metal bioaccumulation and the assessment of potential health risks in different tissues of three fish species from Edremit Bay (Aegean Sea, Turkey). BSAF values were notable mainly for Hg, Zn, Cd and Cu in the liver and, to a lesser extent, in the gills, whereas the lowest values were mostly found in the muscle tissues of the fish species [68]. Adani et al. estimated the BWAF and BSAF of different metals using a pelagic fish and a benthic fish, respectively, in the coastal waters of Kalpakkam (southeast coast of India). The highest BWAF value was obtained for Cu, and for Zn in the case of BSAF. These data showed that these metals tend to accumulate more in benthic species [87]. Monier et al. determined the BWAF and BSAF in the liver and muscle of three fish species from the Damietta Port (North Egypt) on the Mediterranean coast. In general, the order in both tissues for the BWAF values was as follows: Zn > Cu > Pb > Cd, while the BSAF were much lower following this trend: Cu > Cd > Zn ≈ Pb [17].

The concentration of heavy metals in the different fish species and tissues varies depending on several factors, such as the aquatic ecosystem, bioavailability, habitat, life cycle, feeding nature or physiological conditions. Thus, to determine these concentrations and bioaccumulation in different fish species is essential for assessing the risk to the environment and human health [17,40]. Some studies have reported that gills and the liver accumulate higher metal concentrations compared to muscle. The uptake of trace metals through water involves the direct transfer of these elements from the water to the gills and body surfaces [13]. The high levels of heavy metals (such as Cu or Zn) in liver tissue are related to the detoxifying function of this organ, which is the site of metal metabolism [88]. Metallothioneins are proteins in hepatic tissues that act as essential metal stores to fulfil several enzymatic and metabolic demands. The presence of Cd can be explained by the capacity of this metal to displace essential metals normally associated with metallothioneins in hepatic tissues [89]. According to several studies, Cd is one of the most concentrated metals in benthic organisms since molluscs accumulate the most Cd, and they have been identified as pollution indicators. Lead and cadmium have the highest biomagnification levels from prey to predator. In contrast, copper has the least sign of biomagnification in the food chain [20,23]. It has been also reported that demersal fish and benthic communities generally have higher concentrations of arsenic, cadmium, chromium, copper, and zinc [90], which is consistent with the results of this study.

### 3.3. Correlation among Fish, Water and Sediment Metal Contents

Spearman correlation tests (*p* < 0.05) were performed to determine the relationship between metal concentrations in the different compartments. Correlations were found in black scorpionfish species: a considerable positive correlation for Zn between the metal content in the gills and the exchangeable fraction of the sediments (R_Spearman_ = 0.67363) and the metal concentration in the liver and the reducible fraction of sediments (R_Spearman_ = 0.7233); and, on the other hand, negative correlations between the gills and the oxidisable and residual fractions of the sediments (R_Spearman_ = −0.72849 and −0.71005, respectively), explained by the lower availability of these fractions. The correlation between the Zn content in the gills and the exchangeable fraction can be explained because this is the fraction that is most easily released into the environment, and the gills are the first organ in contact with water. In the liver, the release of Zn bound to Fe and Mn (hydr)oxides could also affect these fish species. No more significant correlations were found for the rest of the fish species and water or sediments. However, high concentrations of metals were found in several samples, revealing the influence of anthropogenic activities in the bay.

## 4. Conclusions

The total metal contents of Zn, Cd, Pb, and Cu were determined in the gills, liver, and muscle of the following four fish species common in Algeciras Bay: the benthic species sole (*S. senegalensis*) and the benthopelagic species black scorpionfish (*S. porcus*), streaked gurnard (*T. lastoviza*), and white seabream (*D. sargus sargus*). The trend in metal content was Zn ≥ Cu > Pb ≥ Cd, and for each tissue Zn > Cu ≈ Pb > Cd (gills), Cu ≈ Zn > Pb ≈ Cd (liver), and Zn > Cu > Pb ≈ Cd (muscle). The highest levels of Zn and Cu were found mainly in the liver and gills from sites characterised by anthropogenic activities. Compared to guideline values, percentages of 13%, 27%, 19%, and 20% of the muscle samples of sole, black scorpionfish, streaked gurnard, and white seabream, respectively, exceeded the most restrictive guideline value for Pb (FAO value: 0.11 mg/kg d.w.). Therefore, the consumption of these fish species may pose a potential risk to human health in relation to Pb. On the other hand, MPI values revealed that the liver and gills of benthic species were more affected by metal contamination than benthopelagic species, especially at most industrialised sites. Regarding the bioaccumulation factors, it can be stated that the liver of the sole species presented a high bioaccumulation of Cd and Cu from the sediment (BSAF). These benthic fish were the most affected by the sediments in this area. From all the studies accomplished, it could be concluded that, among the fish species studied, *S. senegalensis* and *D. sargus sargus* can be considered representative and useful pollution bioindicators.

All these results were consistent with the findings in *Ecological Status of Algeciras Bay, in a Highly Anthropised Area in South-West Europe, through Metal Assessment—Part I: Abiotic Samples*: elevated Zn levels in water, indicative of anthropogenic influence; a notable percentage of dissolved Cd, the most available form; higher Pb levels in water compared to other affected ecosystems; the highest metal contents in sediments at sites 3 and 4, exceeding the TEL for Cu and nearing it for Pb; and substantial availabilities of Cd (fraction F1) and Pb (F1 + F2) in sediments and their potential resuspension into the water column, impacting fish species.

These integrative environmental studies have effectively revealed Algeciras Bay’s ecological status, highlighting potential impacts from industrial, maritime, and urban on the aquatic ecosystem and biota. Hence, the control of Zn, Cd, Pb, and Cu pollution and monitoring of fish and seafood for human consumption would be advisable in this area.

## Figures and Tables

**Table 1:** Ranges and average metal concentrations (mg/kg d.w.) for fish tissues at each sampling site.

Site	Tissue	Zn	Cd	Pb	Cu
1 Getares beach	Gills (*n* = 40)	(6–1153) 98 ± 196	(<LD) <LD	(<LD–39.1) 3.05 ± 7.35	(0.6–10.8) 3.22 ± 1.60
	Liver (*n* = 33)	(17–503) 121 ± 86	(0.06–2.90) 0.96 ± 0.70	(<LD–7.71) 2.76 ± 2.05	(4–1492) 313 ± 335
	Muscle (*n* = 40)	(12.3–43.8) 20.1 ± 7.5	(<LD) <LD	(<LD–3.13) <LD ^a^	(0.28–1.98) 0.82 ± 0.37
2 Isla Verde	Gills (*n* = 9)	(38.5–80.7) 61.2 ± 12.0	(<LD–0.21) <LD	(<LD–4.63) 1.25 ± 1.45	(1.20–3.78) 2.45 ± 0.93 ^b^
	Liver (*n* = 7)	(72–475) 207 ± 137	(0.23–3.83) 1.36 ± 1.55	(<LD–2.06) 0.73 ± 0.81	(9–636) 104 ± 234
	Muscle (n = 8)	(12.5–46.6) 24.0 ± 11.5	(<LD) <LD	(<LD) <LD	(0.30–2.02) 0.74 ± 0.67
3 Palmones	Gills (*n* = 18)	(<LD–74.30) 65.8 ± 17.4	(<LD–0.10) <LD	(<LD–7.06) 2.14 ± 1.78	(<LD–31.83) 4.37 ± 8.23
	Liver (*n* = 18)	(68–250) 127 ± 44	(<LD–2.53) 0.38 ± 0.58	(<LD–5.12) 1.03 ± 1.39	(10–426) 92 ± 132
	Muscle (*n* = 21)	(<LD–260) 30.0 ± 53.1	(<LD–0.40) <LD	(<LD–1.06) <LD	(<LD–2.48) 1.17 ± 0.64 ^c^
4 Guadarranque	Gills (*n* = 12)	(58–111) 69.7 ± 19.5	(<LD–0.11) <LD	(<LD–2.31) 0.76 ± 0.74	(1.39–9.87) 2.91 ± 2.28
	Liver (*n* = 11)	(14–299) 139 ± 90	(<LD–1.73) 0.79 ± 0.51	(<LD–2.56) 0.88 ± 0.96	(7–262) 69.1 ± 89.6
	Muscle (*n* = 11)	(11.7–41.5) 22.0 ± 8.0	(<LD–0.10) <LD	(<LD–1.49) 0.20 ± 0.45	(0.4–10.1) 1.55 ± 2.86
5 Puente Mayorga	Gills (*n* = 8)	(<LD–151) 67.7 ± 41.4	(<LD–0.52) <LD	(0.51–8.20) 2.58 ± 3.06	(2.1–34.1) 7.4 ± 10.8
	Liver (*n* = 7)	(63–127) 104 ± 27	(<LD–1.22) 0.47 ± 0.48	(<LD–7.56) 2.35 ± 2.90	(2–1505) 268 ± 548
	Muscle (*n* = 7)	(16.3–25.5) 19.5 ± 3.0	(<LD) <LD	(<LD–0.29) <LD	(0.57–2.10) 1.03 ± 0.53

^a^ *n* = 39; ^b^ *n* = 8; ^c^ *n* = 20.

**Table 2:** Permissible levels of metals in muscle meat of fish found in the literature and this study (referred to mg/kg of dry weight).

Organisation ^a^	Zn	Cd	Pb	Cu	Reference
FAO maximum limit for fish	30–100 (143–476) ^b^	0.05–5.5 (0.24–26.2) ^b^	0.5–6.0 (0.11–28.6) ^b^	10–100 (47.6–476) ^b^	[41]
WHO 1989	100 (476) ^b^	1 (4.8) ^b^	2 (9.6) ^b^	30 (143) ^b^	[42]
England MAFF	50 (238) ^b^	0.2 (0.96) ^b^	2 (9.6) ^b^	20 (95.2) ^b^	[43]
EC, EU		0.05 (0.24) ^b^	0.3 (1.4) ^b^		[44,45,46]
TFC	50 (238) ^b^	0.1 (0.48) ^b^	1 (4.8) ^b^	20 (95.2) ^b^	[47]
**Algeciras Bay**	**Zn**	**Cd**	**Pb**	**Cu**	**Reference**
Minimum-maximum values					This study
sole	<LD–32.3	<LD–0.1	<LD–0.35	<LD–10.1	
black scorpionfish	14.0–41.5	<LD	<LD–3.1	0.3–2.0	
streaked gurnard	12–260	<LD–0.4	<LD–1.5	0.4–2.5	
white seabream	16.0–46.6	<LD	<LD–0.9	0.3–1.3	
Percentage surpassing the most restrictive FAO limits ^c^					
sole	0%	0%	13%	0%	
black scorpionfish	0%	0%	27%	0%	
streaked gurnard	5%	5%	19%	0%	
white seabream	0%	0%	20%	0%	

^a^ FAO: Food and Agriculture Organisation of the United Nations, WHO: World Health Organisation, MAFF: Ministry of Agriculture, Forestry and Fisheries, EC: European Commission, EU: European Union, TFC: Turkish Food Codex; ^b^ Conversion from wet to dry weight (conversion factor of 0.208 considering 79% of moisture content) [17]; ^c^ Zn: 143, Cd: 0.24, Pb: 0.11, Cu: 47.6 mg/kg of dry weight.

**Table 3:** Comparison of average values of total metal concentrations (mg/kg d.w.) in fish species with other studies ^a^.

Tissue	Zn	Cd	Pb	Cu	Site	Reference
*Sole*						
Gills	58.5	<LD	2.76	5.29	Algeciras Bay (Spain)	This study
Liver	98.7	0.84	2.95	356		
Muscle	17.5	<LD	<LD	0.98		
Gills	73.7 (−1.3)	0.56	4.52 (−1.6)	11.9 (−2.2)	Huelva Estuary (Spain)	[35]
Liver	88.2 (+1.1)	1.82 (−2.2)	1.46 (+2.0)	433.5 (−1.2)		
Muscle	23.7 (−1.4)	0.01	0.4	1.41 (−1.4)		
Gills	94.5 (−1.6)	0.4	2.72 (1.0)	5.12 (1.0)	Cádiz Bay (Spain)	[48]
Liver	120.8 (−1.2)	0.55 (+1.5)	0.086 (+34.3)	441.6 (−1.2)		
Muscle	26.1 (−1.5)	0.1	0.01	0.78 (+1.3)		
Liver	78.0 (+1.3)	0.7 (+1.2)	0.14 (+20.9)	426.5 (−1.2)	Senegalese coasts (Africa)	[49]
Muscle	17.4 (1.0)	<LD	<LD	0.49 (+2.0)		
*Black scorpionfish*						
Gills	68.2	0.014	0.76	2.28	Algeciras Bay (Spain)	This study
Liver	223.7	1.01	0.20	17.6		
Muscle	26.3	<LD	0.32	0.98		
Muscle	43.2 (−1.6)	0.024	0.026 (+12.1)	0.56 (+1.7)	Northwestern Mediterranean Sea (France)	[50]
Muscle	4.37 (+6.0)	0.002	0.013 (+24.9)	0.22 (+4.5)	Tuscany coast (Italy)	[51]
Muscle	2.40 (+11.0)	0.01	0.02 (+15.9)	0.10 (+9.8)	Black Sea (Turkey)	[52]
Gills		<LD	0.04 (+19.1)	0.06 (+38.0)	Black Sea (Turkey)	[53]
Liver		<LD	0.03 (+6.7)	0.45 (+39.2)		
Muscle		0.02	0.04 (+7.9)	0.07 (+14.0)		
Muscle		0.001	0.04 (+7.8)		Cassidaigne Canyon (France)	[54]
Muscle	95.3 (−3.6)	0.80	0.66 (−2.1)	0.73 (+1.3)	Black and Aegean Seas (Turkey)	[55]
Muscle	10 (+2.6)		0.5 (−1.6)		Augusta Bay (Italy)	[56]
*White seabream*						
Gills	198.5	<LD	6.87	2.23	Algeciras Bay (Spain)	This study
Liver	113.2	1.50	2.09	133.9		
Muscle	32.5	<LD	0.19	0.88		
Liver	83.0 (+1.4)	2.67 (−1.8)	0.21 (+10.0)	23.7 (+5.7)	Gran Canaria (Canary Islands, Spain)	[57]
Muscle	4.51 (+7.2)	0.003	0.02 (+11.0)	0.57 (+1.5)		
Liver		3.15 (−2.1)	0.68 (+3.1)	44.4 (+3.0)	Ria Formosa (Portugal)	[58]
Muscle		0.005	0.036 (+5.2)	1.52 (−1.7)		
Muscle	17.3 (+1.9)	1.48	10.6 (−56.7)	2.91 (−3.3)	Seixal Bay (Portugal)	[59]
Muscle	28.8 (+1.1)		0.11 (+1.8)	2.25 (−2.6)	Bay of Toulon (France)	[60]
Muscle		0.001	0.014 (+13.4)		Cassidaigne Canyon (France)	[54]
Muscle	46.1 (−1.4)	0.11	1.99 (−10.6)	1.53 (−1.7)	Cádiz Bay (Spain)	[61]

^a^ Positive/negative values in red/green mean the times the results of this study are higher/lower compared to the others.

**Table 4:** Comparison of MPI indexes in different tissues of fish species for Zn, Cd, Pb, and Cu with other studies.

Fish Specie		MPI			Site	Reference
		Gills	Liver	Muscle		
*S. senegalensis* (sole)		2.81	15.8	1.34	Algeciras Bay (Spain)	This study
*S. porcus* (black scorpionfish)		1.80	5.21	2.18		
*T. lastoviza* (streaked gurnard)		2.15	4.75	0.73		
*D. sargus sargus* (white seabream)		3.71	12.5	3.41		
*S. senegalensis*		4.79	7.09	0.38	Cádiz Bay (Spain)	[48]
		-	7.55	0.27	Senegalese coast (Africa)	[49]
*S. porcus*		-	-	0.35	Northwestern Mediterranean Sea (France)	[50]
		-	-	0.07	Tuscany coast (Italy)	[51]
		-	-	0.08	Black Sea area	[52]
		-	-	2.46	Black and Aegean Seas (Turkey)	[55]
*D. sargus sargus*		-	-	5.30	Seixal Bay (Portugal)	[59]
		-	-	1.98	Cadiz Bay (Spain)	[61]
*D. sargus cadenati*		-	5.76	0.10	North coast of Gran Canaria (Canary Islands)	[57]
*D. vulgaris*		-	-	0.87	Cádiz Bay (Spain)	[61]
*M. barbatus*		-	-	1.42		
*M. surmuletus*		-	-	0.98		
*P. acarne*		-	-	1.54		
*P. erythrinus*		-	-	1.09		
*P. auriga*		-	-	1.49		
*P. pagrus*		-	-	1.27		
*S. aurata*		-	-	1.17		
*L. aurata*		-	6.37	0.23	Odiel Estuary (Spain)	[63]
*A. anguilla*		-	3.95	0.42		
*S. vulgaris*		-	4.15	0.27		
*L. aurata*		-	2.13	0.16	Cadiz Bay (Spain)	
*A. anguilla*		-	2.39	0.26		
*S. vulgaris*		-	2.02	0.18		
*S. senegalensis*		6.86	17.85	0.60	Ría de Huelva (Spain)	[35]
*S. aurata*		4.35	27.93	0.72		
*L. abu*		1.02	-	0.75	Tigris river (Turkey)	[64]
*C. regium*		0.62	-	0.42		
*C. macrostomus*		0.94	-	0.64		
*B. mystaceus*		0.19	-	0.19		
*C. trutta*		0.13	-	0.16		
*C. gibelio*		0.16	-	0.36		
*G. oyena*		2.19	2.83	0.54	Hurghada City, Red Sea (Egypt)	[65]
*S. sordidus*		1.45	0.75	0.62		
*L. lentjan*		1.62	3.63	0.81		
*S. rivulatus*		1.60	13.61	0.43		
*M. vanicolensis*		2.81	3.35	0.98		
*S. solea*	32.29		80.87	11.53	Iskenderun Gulf (Turkey)	[9]
*S. aurata*	22.81		41.49	9.42		
*L. tanakae*	-		-	0.68	Shandong Peninsula, Yellow Sea (China)	[66]
*O. kenojei*	-		-	0.88		
*C. stigmatias*	-		-	0.56		
*C. joyneri*	-		-	0.57		
*S. schlegelii*	-		-	0.63		
*L. litulon*	-		-	0.53		
*P. polyactis*	-		-	0.92		
*P. indicus*	-		-	0.68		
*L. micropterus*	-		-	0.40		
*S. niphonius*	-		-	0.69		
*K. punctatus*	-		-	0.56		
*M. cephalus*	-		2.97	1.89	Damietta Port (Egypt)	[17]
*P. pagrus*	-		0.97	0.56		
*S. aurita*	-		0.65	0.33		
*M. merluccius*	1.33		5.98	0.36	Edremit Bay, Aegean Sea (Turkey)	[67]
*M. barbatus*	0.99		4.01	0.33		
*P. erythrinus*	1.82		11.25	0.45		

**Table 5:** Number and percentage of fish samples surpassing reference BWAF and BSAF values (most significant results (>50%) in red font).

Tissue	Factor	Values	Zn	Cd	Pb	Cu
*Sole*						
Gills	BWAF	>1	46/46 (100%)	2/4 (50%)	27/43 (63%)	12/13 (92%)
		>100	1/46 (2%)	0/4 (0%)	0/43 (0%)	0/13 (0%)
	BSAF	<1	5/46 (11%)	22/22 (100%)	43/46 (94%)	42/46 (92%)
		1–2	38/46 (83%)	0/22 (0%)	2/46 (4%)	2/46 (4%)
		>2	3/46 (6%)	0/22 (0%)	1/46 (2%)	2/46 (4%)
Liver	BWAF	>1	39/39 (100%)	2/2 (100%)	34/36 (94%)	11/11 (100%)
		>100	8/39 (21%)	0/2 (0%)	0/36 (0%)	**6/11 (55%)**
	BSAF	<1	3/39 (8%)	6/21 (29%)	39/39 (100%)	0/39 (0%)
		1–2	9/39 (23%)	3/21 (14%)	0/39 (0%)	3/39 (8%)
		>2	**27/39 (69%)**	**12/21 (57%)**	0/39 (0%)	**36/39 (92%)**
Muscle	BWAF	>1	45/46 (98%)	0/4 (0%)	0/42 (0%)	5/12 (42%)
		>100	0/46 (0%)	0/4 (0%)	0/42 (0%)	0/12 (0%)
	BSAF	<1	46/46 (100%)	22/22 (100%)	45/45 (100%)	46/46 (100%)
		1–2	0/46 (0%)	0/22 (0%)	0/45 (0%)	0/46 (0%)
		>2	0/46 (0%)	0/22 (0%)	0/45 (0%)	0/46 (0%)
*Black scorpionfish*						
Gills	BWAF	>1	15/15 (100%)	-	8/13 (62%)	1/1 (100%)
		>100	2/15 (13%)	-	0/13 (0%)	0/1 (0%)
	BSAF	<1	2/15 (13%)	15/15 (100%)	15/15 (100%)	14/14 (100%)
		1–2	12/15 (80%)	0/15 (0%)	0/15 (0%)	0/14 (0%)
		>2	1/15 (7%)	0/15 (0%)	0/15 (0%)	0/14 (0%)
Liver	BWAF	>1	12/12 (100%)	-	2/10 (20%)	1/1 (100%)
		>100	**10/12 (83%)**	-	0/10 (0%)	0/1 (0%)
	BSAF	<1	0/12 (0%)	2/12 (17%)	12/12 (100%)	8/12 (67%)
		1–2	1/12 (8%)	1/12 (8%)	0/12 (0%)	3/12 (25%)
		>2	**11/12 (92%)**	**9/12 (75%)**	0/12 (0%)	1/12 (8%)
Muscle	BWAF	>1	15/15 (100%)	-	3/13 (23%)	0/1 (0%)
		>100	0/15 (0%)	-	0/13 (0%)	0/1 (0%)
	BSAF	<1	15/15 (100%)	15/15 (100%)	15/15 (100%)	15/15 (100%)
		1–2	0/15 (0%)	0/15 (0%)	0/15 (0%)	0/15 (0%)
		>2	0/15 (0%)	0/15 (0%)	0/15 (0%)	0/15 (0%)
*Streaked gurnard*						
Gills	BWAF	>1	19/21 (91%)	0/12 (0%)	16/18 (89%)	1/1 (100%)
		>100	1/21 (5%)	0/12 (0%)	0/18 (0%)	0/1 (0%)
	BSAF	<1	14/21 (67%)	20/20 (100%)	21/21 (100%)	21/21 (100%)
		1–2	6/21 (28%)	0/20 (0%)	0/21 (0%)	0/21 (0%)
		>2	1/21 (5%)	0/20 (0%)	0/21 (0%)	0/21 (0%)
Liver	BWAF	>1	20/20 (100%)	9/12 (75%)	9/17 (53%)	2/2 (100%)
		>100	3/20 (15%)	0/12 (0%)	0/17 (0%)	0/2 (0%)
	BSAF	<1	1/20 (5%)	6/19 (31.5%)	20/20 (100%)	12/20 (60%)
		1–2	13/20 (65%)	7/19 (37%)	0/20 (0%)	4/20 (20%)
		>2	6/20 (30%)	6/19 (31.5%)	0/20 (0%)	4/20 (20%)
Muscle	BWAF	>1	21/21 (100%)	1/14 (7%)	2/18 (11%)	1/1 (100%)
		>100	0/21 (0%)	0/14 (0%)	0/18 (0%)	0/1 (0%)
	BSAF	<1	20/21 (95%)	19/20 (95%)	21/21 (100%)	20/20 (100%)
		1–2	0/21 (0%)	0/20 (0%)	0/21 (0%)	0/20 (0%)
		>2	1/21 (5%)	1/20 (5%)	0/21 (0%)	0/20 (0%)
*White seabream*						
Gills	BWAF	>1	5/5 (100%)	-	3/3 (100%)	1/1 (100%)
		>100	1/5 (20%)	-	0/3 (0%)	0/1 (0%)
	BSAF	<1	0/5 (0%)	3/3 (100%)	5/5 (100%)	5/5 (100%)
		1–2	1/5 (20%)	0/3 (0%)	0/5 (0%)	0/5 (0%)
		>2	**4/5 (80%)**	0/3 (0%)	0/5 (0%)	0/5 (0%)
Liver	BWAF	>1	5/5 (100%)	-	2/3 (67%)	1/1 (100%)
		>100	2/5 (40%)	-	0/3 (0%)	0/1 (0%)
	BSAF	<1	0/5 (0%)	0/3 (0%)	5/5 (100%)	1/5 (20%)
		1–2	0/5 (0%)	1/3 (33%)	0/5 (0%)	2/5 (40%)
		>2	**5/5 (100%)**	**2/3 (67%)**	0/5 (0%)	2/5 (40%)
Muscle	BWAF	>1	5/5 (100%)	-	1/3 (33%)	0/1 (0%)
		>100	0/5 (0%)	-	0/3 (0%)	0/1 (0%)
	BSAF	<1	3/5 (60%)	3/3 (100%)	5/5 (100%)	5/5 (100%)
		1–2	2/5 (40%)	0/3 (0%)	0/5 (0%)	0/5 (0%)
		>2	0/5 (0%)	0/3 (0%)	0/5 (0%)	0/5 (0%)

## Data Availability

Data is contained within the article or Supplementary Materials.

## Supplementary Materials

The following supporting information can be downloaded at: https://www.mdpi.com/article/10.3390/toxics12030166/s1, Table S1: Geographical coordinates of sampling sites; Table S2: Analytical instruments and equipment; Table S3: Method detection limits of metal analysis; Table S4: Recoveries of the CRMs used for the assessment of the methodology accuracy; Table S5: List of fish samples analysed, biota-water accumulation factor (BWAF) and biota-sediment accumulation factor (BSAF) data.

## References

[1] Karayakar F., Işık U., Cicik B. (2022). Heavy metal levels in economically important fish species sold by fishermen in Karatas (Adana/TURKEY). J. Food Compos. Anal..

[2] Reksten A.M., Rahman Z., Kjellevold M., Gamarro E.G., Thilsted S.H., Pincus L.M., Aakre I., Ryder J., Ariyawansa S., Nordhagen A. (2021). Metal contents in fish from the Bay of Bengal and potential consumer exposure—The EAF-nansen programme. Foods.

[3] Pupavac S.M., Jovanović G.K., Linšak Ž., Glad M., Traven L., Žeželj S.P. (2022). The influence on fish and seafood consumption, and the attitudes and reasons for its consumption in the Croatian population. Front. Sustain. Food Syst..

[4] Altiok S., Murthy A., Iha K., Galli A. (2021). Reducing Mediterranean Seafood Footprints: The role of consumer attitudes. Ocean Coast. Manag..

[5] Frías-Espericueta M.G., Bautista-Covarrubias J.C., Osuna-Martínez C.C., Delgado-Alvarez C., Bojórquez C., Aguilar-Juárez M., Roos-Muñoz S., Osuna-López I., Páez-Osuna F. (2022). Metals and Oxidative Stress in Aquatic Decapod Crustaceans: A Review with Special Reference to Shrimp and Crabs. Aquat. Toxicol..

[6] Zaynab M., Al-Yahyai R., Ameen A., Sharif Y., Ali L., Fatima M., Khan K.A., Li S. (2022). Health and Environmental Effects of Heavy Metals. J. King Saud Univ.-Sci..

[7] Jayaprakash M., Senthil Kumar R., Giridharan L., Sujitha S.B., Sarkar S.K., Jonathan M.P. (2015). Bioaccumulation of Metals in Fish Species from Water and Sediments in Macrotidal Ennore Creek, Chennai, SE Coast of India: A Metropolitan City Effect. Ecotoxicol. Environ. Saf..

[8] Jonathan M.P., Aurioles-Gamboa D., Villegas L.E.C., Bohórquez-Herrera J., Hernández-Camacho C.J., Sujitha S.B. (2015). Metal Concentrations in Demersal Fish Species from Santa Maria Bay, Baja California Sur, Mexico (Pacific Coast). Mar. Pollut. Bull..

[9] Aytekin T., Kargın D., Çoğun H.Y., Temiz Ö., Varkal H.S., Kargın F. (2019). Accumulation and Health Risk Assessment of Heavy Metals in Tissues of the Shrimp and Fish Species from the Yumurtalik Coast of Iskenderun Gulf, Turkey. Heliyon.

[10] Kazemi A., Esmaeilbeigi M., Ansari A., Asl A.G., Mohammadzadeh B. (2022). Alterations and Health Risk Assessment of the Environmental Concentration of Heavy Metals in the Edible Tissue of Marine Fish (Thunnus Tonggol) Consumed by Different Cooking Methods. Reg. Stud. Mar. Sci..

[11] Rajeshkumar S., Li X. (2018). Bioaccumulation of Heavy Metals in Fish Species from the Meiliang Bay, Taihu Lake, China. Toxicol. Rep..

[12] Kalantzi I., Mylona K., Pergantis S.A., Coli A., Panopoulos S., Tsapakis M. (2019). Elemental Distribution in the Different Tissues of Brood Stock from Greek Hatcheries. Aquaculture.

[13] Vetsis E., Kalantzi I., Pergantis S.A., Kokokiris L., Karakassis I. (2021). Metals in Tissues of Marine Fish from the Thermaikos Gulf, Eastern Mediterranean Sea: Detection of Changes with Trophic Level. Mar. Pollut. Bull..

[14] Pan B., Wang Y., Li D., Wang T., Du L. (2022). Tissue-Specific Distribution and Bioaccumulation Pattern of Trace Metals in Fish Species from the Heavily Sediment-Laden Yellow River, China. J. Hazard. Mater..

[15] Ali H., Khan E., Ilahi I. (2019). Environmental Chemistry and Ecotoxicology of Hazardous Heavy Metals: Environmental Persistence, Toxicity, and Bioaccumulation. J. Chem..

[16] Bakhshalizadeh S., Mora-Medina R., Fazio F., Parrino V., Ayala-Soldado N. (2022). Determination of the Heavy Metal Bioaccumulation Patterns in Muscles of Two Species of Mullets from the Southern Caspian Sea. Animals.

[17] Monier M.N., Soliman A.M., Al-Halani A.A. (2023). The Seasonal Assessment of Heavy Metals Pollution in Water, Sediments, and Fish of Grey Mullet, Red Seabream, and Sardine from the Mediterranean Coast, Damietta, North Egypt. Reg. Stud. Mar. Sci..

[18] Allaby M. (2020). A Dictionary of Zoology (Oxford Quick Reference).

[19] Rahman M.S., Akther S., Ahmed A.S.S., Saha N., Rahman L.S., Ahmed M.K., Arai T., Idris A.M. (2022). Distribution and source apportionment of toxic and trace elements in some benthic and pelagic coastal fish species in Karnaphuli River Estuary, Bangladesh: Risk to human health. Mar. Pollut. Bull..

[20] Chan W.S., Routh J., Luo C., Dario M., Miao Y., Luo D., Wei L. (2021). Metal Accumulations in Aquatic Organisms and Health Risks in an Acid Mine-Affected Site in South China. Environ. Geochem. Health.

[21] Monikh F.A., Safahieh A., Savari A., Ronagh M.T., Doraghi A. (2013). The Relationship between Heavy Metal (Cd, Co, Cu, Ni and Pb) Levels and the Size of Benthic, Benthopelagic and Pelagic Fish Species, Persian Gulf. Bull. Environ. Contam. Toxicol..

[22] Man X., Huang H., Chen F., Gu Y., Liang R., Wang B., Jordan R.W., Jiang S. (2022). Anthropogenic Impacts on the Temporal Variation of Heavy Metals in Daya Bay (South China). Mar. Pollut. Bull..

[23] Anagha B., Athira P.S., Anisha P., Charles P.E., Anandkumar A., Rajaram R. (2022). Biomonitoring of Heavy Metals Accumulation in Molluscs and Echinoderms Collected from Southern Coastal India. Mar. Pollut. Bull..

[24] Ivanina A.V., Sokolova I.M. (2015). Interactive Effects of Metal Pollution and Ocean Acidification on Physiology of Marine Organisms. Curr. Zool..

[25] Layglon N., Abdou M., Massa F., Castellano M., Bakker E., Povero P., Tercier-Waeber M.-L. (2022). Speciation of Cu, Cd, Pb and Zn in a Contaminated Harbor and Comparison to Environmental Quality Standards. J. Environ. Manag..

[26] Morillo J., Usero J., Gracia I. (2007). Potential Mobility of Metals in Polluted Coastal Sediments in Two Bays of Southern Spain. J. Coast. Res..

[27] Sánchez-Garrido J.C., Lafuente J.G., Sammartino S., Naranjo C., de los Santos F.J., Álvarez Fanjul E. (2014). Meteorologically-Driven Circulation and Flushing Times of the Bay of Algeciras, Strait of Gibraltar. Mar. Pollut. Bull..

[28] Instituto Nacional de Estadística Cifras Oficiales de Población de los Municipios Españoles en Aplicación de la Ley de Bases del Régimen Local (Art. 17). https://www.ine.es/jaxiT3/Tabla.htm?t=2864&L=0.

[29] Epdata Gibraltar—Cuántos Habitantes Tiene el País, Datos Demográficos. https://www.epdata.es/datos/habitantes-pais-poblacion-datos-estadisticas-demograficos/670/gibraltar/119.

[30] Araújo C.V.M., Diz F.R., Tornero V., Lubián L.M., Blasco J., Moreno-Garrido I. (2010). Ranking Sediment Samples from Three Spanish Estuaries in Relation to Its Toxicity for Two Benthic Species: The Microalga Cylindrotheca Closterium and the Copepod Tisbe Battagliai. Environ. Toxicol. Chem..

[31] Morillo J., Usero J. (2008). Trace Metal Bioavailability in the Waters of Two Different Habitats in Spain: Huelva Estuary and Algeciras Bay. Ecotoxicol. Environ. Saf..

[32] Periáñez R. (2012). Modelling the Environmental Behaviour of Pollutants in Algeciras Bay (South Spain). Mar. Pollut. Bull..

[33] García Sarasa C. (2001). Especies de Interés Pesquero en el Litoral de Andalucía: Vertebrados.

[34] Kosore C.M., Galindo-Riaño M.D., Díaz-de-Alba M. (2015). Assessing Trace-Element Mobility in Algeciras Bay (Spain) Sediments by Acid and Complexing Screening. Arab. J. Chem..

[35] Vicente-Martorell J.J., Galindo-Riaño M.D., García-Vargas M., Granado-Castro M.D. (2009). Bioavailability of Heavy Metals Monitoring Water, Sediments and Fish Species from a Polluted Estuary. J. Hazard. Mater..

[36] Arain M.B., Kazi T.G., Jamali M.K., Baig J.A., Afridi H.I., Jalbani N., Sarfraz R.A. (2009). Comparison of Different Extraction Approaches for Heavy Metal Partitioning in Sediment Samples. Pedosphere.

[37] McGeer J.C., Brix K.V., Skeaff J.M., Deforest D.K., Brigham S.I., Adams W.J., Green A. (2003). Inverse Relationship between Bioconcentration Factor and Exposure Concentration for Metals: Implications for Hazard Assessment of Metals in the Aquatic Environment. Environ. Toxicol. Chem..

[38] Merciai R., Guasch H., Kumar A., Sabater S., García-Berthou E. (2014). Trace metal concentration and fish size: Variation among fish species in a Mediterranean river. Ecotoxicol. Environ. Saf..

[39] Bernet D., Schmidt H., Burkhardt-Holm P., Wahli T. (1999). Histopathology in fish: Proposal for a protocol to assess aquatic pollution. J. Fish Dis..

[40] Varol M., Kaçar E., Sünbül M.R., Towfiqul Islam A.R.M. (2022). Species, Tissue and Gender-Related Metal and Element Accumulation in Fish Species in a Large Reservoir (Turkey) and Health Risks and Nutritional Benefits for Consumers. Environ. Toxicol. Pharmacol..

[41] Mokhtar M.B., Aris A.Z., Munusamy V., Praveena S.M. (2009). Assessment level of heavy metals in *Penaeus monodon* and *Oreochromis* spp. in selected aquaculture ponds of high densities development area. Eur. J. Sci. Res..

[42] FAO/WHO (1989). Evaluation of Certain Food Additives and the Contaminants Mercury, Lead and Cadmium.

[43] MAFF (Ministry of Agriculture, Fisheries and Food) (1998). Monitoring and surveillance of non-radioactive contaminants in the aquatic environment and activities regulating the disposal of wastes at sea. Aquatic Environment Monitoring Report No. 52.

[44] EC (European Community) (2006). Commission Regulation No. 1881/2006 of 19 December 2006 setting maximum levels for certain contaminants in foodstuffs. Off. J. Eur. Union.

[45] EU (2014). Commission regulation (EU) No. 488/2014 of 12 May 2014 amending regulation (EC) No. 1881/2006 as regards maximum levels of cadmium in foodstuffs. L 138/75. Off. J. Eur. Union..

[46] EU (2015). Commission regulation (EU) No. 2015/1005 of 25 June 2015 amending regulation (EC) No. 1881/2006 as regards maximum levels of lead in certain foodstuffs. L 161/9. Off. J. Eur. Union..

[47] Dural M., Lugal Göksu M.Z., Özak A.A., Derici B. (2006). Bioaccumulation of some heavy metals in different tissues of *Dicentrarchus Labrax* L, 1758, *Sparus Aurata* L, 1758 and *Mugil cephalus* L, 1758 from the ÇamlIk lagoon of the eastern cost of Mediterranean (Turkey). Environ. Monit. Assess..

[48] Galindo M.D., Jurado J.A., García M., González de Canales M.L., Oliva M., López F., Granado M.D., Espada E. (2012). Trace Metal Accumulation in Tissues of Sole (*Solea senegalensis*) and the Relationships with the Abiotic Environment. Int. J. Environ. Anal. Chem..

[49] Diop M., Howsam M., Diop C., Cazier F., Goossens J.F., Diouf A., Amara R. (2016). Spatial and Seasonal Variations of Trace Elements Concentrations in Liver and Muscle of Round Sardinelle (*Sardinella Aurita*) and Senegalese Sole (*Solea Senegalensis*) along the Senegalese Coast. Chemosphere.

[50] Ourgaud M., Ruitton S., Bourgogne H., Bustamante P., Churlaud C., Guillou G., Lebreton B., Harmelin-Vivien M.L. (2018). Trace Elements in a Mediterranean Scorpaenid Fish: Bioaccumulation Processes and Spatial Variations. Prog. Oceanogr..

[51] Bonsignore M., Salvagio Manta D., Mirto S., Quinci E.M., Ape F., Montalto V., Gristina M., Traina A., Sprovieri M. (2018). Bioaccumulation of Heavy Metals in Fish, Crustaceans, Molluscs and Echinoderms from the Tuscany Coast. Ecotoxicol. Environ. Saf..

[52] Bat L., Öztekin A., Şahin F. (2018). Heavy Metal Detection in Scorpaena Porcus Linnaeus, 1758 from Sinop Coast of the Black Sea and Potential Risks to Human Health. Curr. Agric. Res. J..

[53] Çulha S.T., Yabanlı M., Baki B., Yozukmaz A. (2016). Heavy Metals in Tissues of Scorpionfish (*Scorpaena porcus*) Caught from Black Sea (Turkey) and Potential Risks to Human Health. Environ. Sci. Pollut. Res..

[54] Bouchoucha M., Chekri R., Leufroy A., Jitaru P., Millour S., Marchond N., Chafey C., Testu C., Zinck J., Cresson P. (2019). Trace Element Contamination in Fish Impacted by Bauxite Red Mud Disposal in the Cassidaigne Canyon (NW French Mediterranean). Sci. Total Environ..

[55] Uluozlu O.D., Tuzen M., Mendil D., Soylak M. (2007). Trace Metal Content in Nine Species of Fish from the Black and Aegean Seas, Turkey. Food Chem..

[56] Scopelliti G., Di Leonardo R., Tramati C.D., Mazzola A., Vizzini S. (2015). Premature Aging in Bone of Fish from a Highly Polluted Marine Area. Mar. Pollut. Bull..

[57] Afonso A., Gutiérrez Á.J., Lozano G., González-Weller D., Lozano-Bilbao E., Rubio C., Caballero J.M., Revert C., Hardisson A. (2018). Metals in *Diplodus sargus cadenati* and *Sparisoma cretense*—A Risk Assessment for Consumers. Environ. Sci. Pollut. Res..

[58] Ferreira M., Caetano M., Costa J., Pousão-Ferreira P., Vale C., Reis-Henriques M.A. (2008). Metal Accumulation and Oxidative Stress Responses in, Cultured and Wild, White Seabream from Northwest Atlantic. Sci. Total Environ..

[59] Caçador I., Costa J.L., Duarte B., Silva G., Medeiros J.P., Azeda C., Castro N., Freitas J., Pedro S., Almeida P.R. (2012). Macroinvertebrates and Fishes as Biomonitors of Heavy Metal Concentration in the Seixal Bay (Tagus Estuary): Which Species Perform Better?. Ecol. Indic..

[60] Bouchoucha M., Brach-Papa C., Gonzalez J.L., Lenfant P., Darnaude A.M. (2018). Growth, Condition and Metal Concentration in Juveniles of Two Diplodus Species in Ports. Mar. Pollut. Bull..

[61] Guerra-García J.M., Baeza-Rojano E., Cabezas M.P., Díaz-Pavón J.J., Pacios I., García-Gómez J.C. (2009). The Amphipods *Caprella penantis* and *Hyale schmidtii* as Biomonitors of Trace Metal Contamination in Intertidal Ecosystems of Algeciras Bay, Southern Spain. Mar. Pollut. Bull..

[62] Usero J., González-Regalado E., Gracia I. (1997). Trace Metals in the Bivalve Molluscs Ruditapes Decussatus and Ruditapes Philippinarum from the Atlantic Coast of Southern Spain. Environ. Int..

[63] Usero J., Izquierdo C., Morillo J., Gracia I. (2003). Heavy metals in fish (*Solea vulgaris*, *Anguilla anguilla* and *Liza aurata*) from salt marshes on the southern Atlantic coast of Spain. Environ. Int..

[64] Töre Y., Ustaoğlu F., Tepe Y., Kalipci E. (2021). Levels of Toxic Metals in Edible Fish Species of the Tigris River (Turkey); Threat to Public Health. Ecol. Indic..

[65] Zaghloul G.Y., Ezz El-Din H.M., Mohamedein L.I., El-Moselhy K.M. (2022). Bio-Accumulation and Health Risk Assessment of Heavy Metals in Different Edible Fish Species from Hurghada City, Red Sea, Egypt. Environ. Toxicol. Pharmacol..

[66] Liu R., Jiang W., Li F., Pan Y., Wang C., Tian H. (2021). Occurrence, partition, and risk of seven heavy metals in sediments, seawater, and organisms from the eastern sea area of Shandong Peninsula, Yellow Sea, China. J. Environ. Manag..

[67] Kontas A., Uluturhan E., Alyuruk H., Darilmaz E., Bilgin M., Altay O. (2020). Metal Contamination in Surficial Sediments of Edremit Bay (Aegean Sea): Spatial Distribution, Source Identification and Ecological Risk Assessment. Reg. Stud. Mar. Sci..

[68] Morais S., Aragão C., Cabrita E., Conceição L.E.C., Constenla M., Costas B., Dias J., Duncan N., Engrola S., Estevez A. (2016). New Developments and Biological Insights into the Farming of Solea Senegalensis Reinforcing Its Aquaculture Potential. Rev. Aquac..

[69] Ghribi R., Correia A.T., Elleuch B., Nunes B. (2019). Testing the Impact of Contaminated Sediments from the Southeast Marine Coast of Tunisia on Biota: A Multibiomarker Approach Using the Flatfish Solea Senegalensis. Environ. Sci. Pollut. Res..

[70] Riba I., Casado-Martínez M.C., Blasco J., DelValls T.A. (2004). Bioavailability of Heavy Metals Bound to Sediments Affected by a Mining Spill Using Solea Senegalensis and Scrobicularia Plana. Mar. Environ. Res..

[71] Salamanca M.J., Jiménez-Tenorio N., Gonzalez de Canales M.L., DelValls T.A. (2008). Determinación de La Toxicidad de Un Vertido de Petróleo Mediante El Uso de Bioensayos Con El Pez Solea Senegalensis. Cienc. Mar..

[72] Solé M., Lima D., Reis-Henriques M.A., Santos M.M. (2008). Stress Biomarkers in Juvenile Senegal Sole, Solea Senegalensis, Exposed to the Water-Accommodated Fraction of the “Prestige” Fuel Oil. Bull. Environ. Contam. Toxicol..

[73] Solé M., Vega S., Varó I. (2012). Characterization of Type “B” Esterases and Hepatic CYP450 Isoenzimes in Senegalese Sole for Their Further Application in Monitoring Studies. Ecotoxicol. Environ. Saf..

[74] Jiménez-Tenorio N., Salamanca M.J., García-Luque E., Gonzalez de Canales M.L., DelValls T.A. (2008). Chronic Bioassay in Benthic Fish for the Assessment of the Quality of Sediments in Different Areas of the Coast of Spain Impacted by Acute and Chronic Oil Spills. Environ. Toxicol..

[75] Costa P.M., Diniz M.S., Caeiro S., Lobo J., Martins M., Ferreira A.M., Caetano M., Vale C., DelValls T.Á., Costa M.H. (2009). Histological Biomarkers in Liver and Gills of Juvenile Solea Senegalensis Exposed to Contaminated Estuarine Sediments: A Weighted Indices Approach. Aquat. Toxicol..

[76] Costa P.M., Caeiro S., Lobo J., Martins M., Ferreira A.M., Caetano M., Vale C., DelValls T.Á., Costa M.H. (2011). Estuarine Ecological Risk Based on Hepatic Histopathological Indices from Laboratory and in Situ Tested Fish. Mar. Pollut. Bull..

[77] Oliva M., José Vicente J., Gravato C., Guilhermino L., Dolores Galindo-Riaño M. (2012). Oxidative Stress Biomarkers in Senegal Sole, Solea Senegalensis, to Assess the Impact of Heavy Metal Pollution in a Huelva Estuary (SW Spain): Seasonal and Spatial Variation. Ecotoxicol. Environ. Saf..

[78] Oliva M., Perales J.A., Gravato C., Guilhermino L., Galindo-Riaño M.D. (2012). Biomarkers Responses in Muscle of Senegal Sole (Solea Senegalensis) from a Heavy Metals and PAHs Polluted Estuary. Mar. Pollut. Bull..

[79] Fonseca V.F., Vasconcelos R.P., Tanner S.E., França S., Serafim A., Lopes B., Company R., Bebianno M.J., Costa M.J., Cabral H.N. (2015). Habitat Quality of Estuarine Nursery Grounds: Integrating Non-Biological Indicators and Multilevel Biological Responses in Solea Senegalensis. Ecol. Indic..

[80] Martins C., Alves de Matos A.P., Costa M.H., Costa P.M. (2015). Alterations in Juvenile Flatfish Gill Epithelia Induced by Sediment-Bound Toxicants: A Comparative in Situ and Ex Situ Study. Mar. Environ. Res..

[81] Briaudeau T., Zorita I., Cuevas N., Franco J., Marigómez I., Izagirre U. (2019). Multi-Annual Survey of Health Status Disturbance in the Bilbao Estuary (Bay of Biscay) Based on Sediment Chemistry and Juvenile Sole (*Solea* spp.) Histopathology. Mar. Pollut. Bull..

[82] La Colla N.S., Botté S.E., Simonetti P., Negrin V.L., Serra A.V., Marcovecchio J.E. (2021). Water, Sediments and Fishes: First Multi Compartment Assessment of Metal Pollution in a Coastal Environment from the SW Atlantic. Chemosphere.

[83] Islam M.S., Ahmed M.K., Habibullah-Al-Mamun M., Masunaga S. (2015). Assessment of Trace Metals in Fish Species of Urban Rivers in Bangladesh and Health Implications. Environ. Toxicol. Pharmacol..

[84] Avigliano E., Monferrán M.V., Sánchez S., Wunderlin D.A., Gastaminza J., Volpedo A.V. (2019). Distribution and Bioaccumulation of 12 Trace Elements in Water, Sediment and Tissues of the Main Fishery from Different Environments of the La Plata Basin (South America): Risk Assessment for Human Consumption. Chemosphere.

[85] Ahmed A.S.S., Rahman M., Sultana S., Babu S.M.O.F., Sarker M.S.I. (2019). Bioaccumulation and Heavy Metal Concentration in Tissues of Some Commercial Fishes from the Meghna River Estuary in Bangladesh and Human Health Implications. Mar. Pollut. Bull..

[86] Rubalingeswari N., Thulasimala D., Giridharan L., Gopal V., Magesh N.S., Jayaprakash M. (2021). Bioaccumulation of Heavy Metals in Water, Sediment, and Tissues of Major Fisheries from Adyar Estuary, Southeast Coast of India: An Ecotoxicological Impact of a Metropolitan City. Mar. Pollut. Bull..

[87] Adani P., Sawale A.A., Nandhagopal G. (2022). Bioaccumulation of Heavy Metals in the Food Components from Water and Sediments in the Coastal Waters of Kalpakkam, Southeast Coast of India. Environ. Nanotechnol. Monit. Manag..

[88] Beg M.U., Al-Jandal N., Al-Subiai S., Karam Q., Husain S., Butt S.A., Ali A., Al-Hasan E., Al-Dufaileej S., Al-Husaini M. (2015). Metallothionein, Oxidative Stress and Trace Metals in Gills and Liver of Demersal and Pelagic Fish Species from Kuwaits’ Marine Area. Mar. Pollut. Bull..

[89] Amiard J.C., Amiard-Triquet C., Barka S., Pellerin J., Rainbow P.S. (2006). Metallothioneins in Aquatic Invertebrates: Their Role in Metal Detoxification and Their Use as Biomarkers. Aquat. Toxicol..

[90] Yi Y., Yang Z., Zhang S. (2011). Ecological Risk Assessment of Heavy Metals in Sediment and Human Health Risk Assessment of Heavy Metals in Fishes in the Middle and Lower Reaches of the Yangtze River Basin. Environ. Pollut..

