# Supplementary material for: Ecological Status of Algeciras Bay, in a Highly Anthropised Area in South-West Europe, through Metal Assessment—Part II: Biotic Samples
Source: Toxics. 2024 Feb 21;12(3):166. doi: 10.3390/toxics12030166 (PMC10974771; doi:10.3390/toxics12030166)
Supplement: Supplementary file 1 [file toxics-12-00166-s001.zip › toxics-2818213-supplementary.pdf]

Supplementary Material

Ecological Status of Algeciras Bay, in a Highly Anthropised Area in South-West Europe, through Metal Assessment—Part II: Biotic Samples

María José Casanueva-Marenco, María Dolores Galindo-Riaño *, María Dolores Granado-Castro and Margarita Díaz-de-Alba

Department of Analytical Chemistry, Institute of Biomolecules (INBIO), Faculty of Sciences, International Campus of Excellence of the Sea (CEI-MAR), University of Cadiz, Campus Rio San Pedro, 11510 Puerto Real, Cadiz, Spain; mariajose.casanueva@uca.es (M.J.C.-M.); dolores.granado@uca.es (M.D.G.-C.); margarita.diaz@uca.es (M.D.-d.-A.)

* Correspondence: dolores.galindo@uca.es; Tel.: +34-956016362

Table S1. Geographical coordinates of sampling sites in Algeciras Bay

Sampling sites	Coordinates	
	**North**	**West**
1 – Getares beach	36° 05´ 28.31´´	5° 26´ 10.93´´
2 – Isla Verde	36° 07´ 8.43´´	5° 25´ 37.60´´
3 – Palmones	36° 10´ 19.51´´	5° 25´ 27.14´´
4 – Guadarranque	36° 10´ 32.21´´	5° 24´ 27.47´´
5 – Puente Mayorga	36° 10´32.23´´	5° 23´ 23.24´´

Table S2. Analytical instruments and equipment used in this study

Instrument and equipment	Specifications
Ultrapure water system	Millipore Milli-Q50 (Millipore, Burlington, Massachusetts, USA) (resistivity of 18.2 MΩ·cm at 25 °C)
Closed Teflon reactor	PTFE, 100 mL (BRAND, 1305 38, Wertheim, Germany)
Vertical laminar flow cabinet (for samples and solutions)	Cruisair 870-FL (Cruma, Saint Boi de Llobregat, Barcelona, Spain)
Fume chamber (for acids and other fuming reagents)	Waldner 1800 (Wangen im Allgäu, Germany)
Freeze dryer	FreeZone Triad 7400030 (Cole-Parmer, Vernon Hills, Illinois, USA)
Microwave assisted digester	Ethos 1600 (Milestone, Sorisole, Italy)
Inductively coupled plasma-mass spectrometer	X-Series ICP-MS equipment (Thermo Elemental, Winsford, UK) [a]

[a] ICP-MS equipment was calibrated using ^{71}Ga, ^{103}Rh and ^{209}Bi as internal standards in order to minimise matrix interference effects.

Table S3. Method detection limits (MDL) (mg/kg) for the analysis of the different samples (n = 8)

Sample / method	Zn	Cd	Pb	Cu
Total content in gills and muscle / (ICP-MS)	0.21	0.001	0.02	0.03
Total content in liver / (ICP-MS)	0.04	0.002	0.01	0.03

Table S4. Recoveries (%) of the CRMs used for the assessment of the accuracy of the methodology (n = 4)

CRM	Recovery (%)			
	**Zn**	**Cd**	**Pb**	**Cu**
DORM-2	98.7 ± 4.4	106 ± 15	103 ± 6	92.9 ± 4.2
DOLT-3	97.0 ± 1.7	97.4 ± 4.5	94.7 ± 6.9	95.4 ± 7.0

Table S5. Characteristics of fish analysed in Algeciras Bay, biota-water accumulation factor (BWAF) and biota-sediment accumulation factor (BSAF)

Sampling	Site	Sample	Specie [a]	Tissue	Weight (g)	Length (cm)	BWAF				BSAF			
							Zn	Cd	Pb	Cu	Zn	Cd	Pb	Cu
1	1	1	*D.s.*	Gills	143	21	67.4	*	8.5	*	23.6	*	0.4	0.4
1	1	1	*D.s.*	Liver	143	21	6.8	*	2.3	*	2.4	*	0.1	93.0
1	1	1	*D.s.*	Muscle	143	21	4.5	*	*	*	1.6	*	*	0.2
1	1	4	*S.s.*	Gills	236	29	4.8	*	0.4	*	1.7	*	0.02	0.6
1	1	4	*S.s.*	Liver	236	29	11.8	*	3.3	*	4.1	*	0.2	115.6
1	1	4	*S.s.*	Muscle	236	29	1.8	*	*	*	0.6	*	*	0.1
1	1	5	*S.s.*	Gills	115	23	6.2	*	0.7	*	2.2	*	0.03	0.6
1	1	5	*S.s.*	Liver	115	23	10.4	*	3.9	*	3.6	*	0.2	54.1
1	1	5	*S.s.*	Muscle	115	23	1.4	*	*	*	0.5	*	*	0.1
1	1	7	*S.s.*	Gills	225	28	5.0	*	*	*	1.8	*	*	0.3
1	1	7	*S.s.*	Liver	225	28	7.3	*	2.2	*	2.6	*	0.1	79.6
1	1	7	*S.s.*	Muscle	225	28	1.7	*	*	*	0.6	*	*	0.1
1	3	1	*T.l.*	Liver	355	32	28.1	0.8	0.4	57.8	1.7	0.2	0.03	2.0
1	3	1	*T.l.*	Muscle	355	32	3.4	*	0.4	1.7	0.2	*	0.03	0.1
1	5	1	*T.l.*	Gills	76	20	*	*	2.7	*	*	*	0.1	0.4
1	5	1	*T.l.*	Liver	359	33	13.6	*	1.0	*	1.8	*	0.02	0.2
1	5	1	*T.l.*	Muscle	76	20	3.5	*	*	*	0.5	*	*	0.1
1	5	2	*S.s.*	Gills	359	33	12.0	*	1.7	*	1.6	*	0.03	0.4
1	5	2	*S.s.*	Liver	359	33	25.5	*	5.1	*	3.4	*	0.1	137.9
1	5	2	*S.s.*	Muscle	359	33	4.3	*	0.6	*	0.6	*	0.01	0.1
2	1	1	*S.s.*	Gills	135	24	7.0	*	23.5	*	1.8	*	2.7	0.6
2	1	1	*S.s.*	Liver	135	24	11.8	*	1.7	*	3.0	*	0.2	132.4
2	1	1	*S.s.*	Muscle	135	24	3.3	*	*	*	0.8	*	*	0.3
2	1	2	*S.s.*	Gills	136	24	6.7	*	0.7	*	1.7	*	0.1	1.0
2	1	2	*S.s.*	Muscle	136	24	1.5	*	*	*	0.4	*	*	0.2
2	1	3	*S.s.*	Gills	114	21	7.5	*	9.4	*	1.9	*	1.1	0.6
2	1	3	*S.s.*	Liver	114	21	11.5	*	3.4	*	2.9	*	0.4	4.7
2	1	3	*S.s.*	Muscle	114	21	2.4	*	*	*	0.6	*	*	0.1
2	1	4	*S.s.*	Liver	170	26	16.0	*	1.7	*	4.0	*	0.2	19.2
2	1	4	*S.s.*	Muscle	170	26	1.5	*	*	*	0.4	*	*	0.1
2	1	5	*S.s.*	Gills	149	25	5.7	*	0.6	*	1.4	*	0.1	0.6
2	1	5	*S.s.*	Gills	149	25	6.9	*	0.4	*	1.7	*	0.04	0.9
2	1	5	*S.s.*	Liver	149	25	14.6	*	1.6	*	3.7	*	0.2	208.5
2	1	5	*S.s.*	Muscle	149	25	1.9	*	*	*	0.5	*	*	0.4
2	1	6	*S.s.*	Gills	153	26	7.3	*	9.2	*	1.8	*	1.1	0.8
2	1	6	*S.s.*	Muscle	153	26	2.5	*	0.2	*	0.6	*	0.02	0.1
2	1	8	*S.s.*	Gills	183	28	6.2	*	0.1	*	1.6	*	0.02	0.7
2	1	8	*S.s.*	Liver	183	28	13.8	*	1.3	*	3.5	*	0.1	345.1
2	1	8	*S.s.*	Muscle	183	28	2.1	*	*	*	0.5	*	*	0.3
2	1	9	*S.s.*	Gills	112	23	6.9	*	0.2	*	1.7	*	0.03	1.0
2	1	9	*S.s.*	Muscle	112	23	1.6	*	*	*	0.4	*	*	0.1
2	1	10	*S.s.*	Gills	130	25	6.3	*	1.3	*	1.6	*	0.2	0.8

2	1	10	*S.s.*	Liver	130	25	12.4	*	2.4	*	3.1	*	0.3	73.5
2	1	10	*S.s.*	Muscle	130	25	2.6	*	*	*	0.7	*	*	0.2
2	1	11	*S.s.*	Gills	134	24	6.2	*	0.3	*	1.6	*	0.03	0.8
2	1	11	*S.s.*	Liver	134	24	12.0	*	1.2	*	3.0	*	0.1	100.3
2	1	11	*S.s.*	Muscle	134	24	1.7	*	*	*	0.4	*	*	0.2
2	1	12	*S.s.*	Gills	274	31	7.4	*	1.0	*	1.9	*	0.1	0.7
2	1	12	*S.s.*	Muscle	274	31	2.2	*	*	*	0.6	*	*	0.1
2	1	13	*S.s.*	Gills	199	28	6.7	*	0.2	*	1.7	*	0.03	0.8
2	1	13	*S.s.*	Liver	199	28	12.0	*	1.3	*	3.0	*	0.1	91.2
2	1	13	*S.s.*	Muscle	199	28	1.9	*	*	*	0.5	*	*	0.2
2	1	14	*D.s.*	Gills	77	8	10.2	*	1.8	*	2.6	*	0.2	0.4
2	1	14	*D.s.*	Liver	77	8	15.9	*	0.6	*	4.0	*	0.1	2.7
2	1	14	*D.s.*	Muscle	77	8	2.0	*	*	*	0.5	*	*	0.3
2	1	15	*S.s.*	Gills	223	29	7.8	*	0.2	*	2.0	*	0.02	0.7
2	1	15	*S.s.*	Liver	223	29	12.3	*	1.7	*	3.1	*	0.2	11.8
2	1	15	*S.s.*	Muscle	223	29	2.5	*	*	*	0.6	*	*	0.1
2	1	17	*S.s.*	Gills	201	28	6.6	*	0.5	*	1.7	*	0.1	0.7
2	1	17	*S.s.*	Liver	201	28	19.6	*	4.2	*	4.9	*	0.5	148.2
2	1	17	*S.s.*	Muscle	201	28	1.7	*	*	*	0.4	*	*	0.1
2	2	3	*S.s.*	Gills	242	29	9.0	1.7	1.8	*	1.8	*	0.04	0.4
2	2	3	*S.s.*	Liver	242	29	18.5	19.3	5.7	*	3.6	*	0.1	72.0
2	2	3	*S.s.*	Muscle	242	29	1.7	*	*	*	0.3	*	*	0.0
2	2	4	*S.s.*	Gills	287	32	7.3	*	*	*	1.4	*	*	0.3
2	2	4	*S.s.*	Muscle	287	32	2.7	*	*	*	0.5	*	*	0.1
2	2	5	*S.s.*	Gills	386	37	8.5	*	2.1	*	1.7	*	0.05	0.3
2	2	5	*S.s.*	Muscle	386	37	1.6	*	*	*	0.3	*	*	0.04
2	3	3	*S.s.*	Gills	197	28	7.0	1.1	6.7	*	0.9	0.6	0.1	2.0
2	3	3	*S.s.*	Liver	197	28	8.3	7.0	15.3	*	1.0	3.7	0.2	21.3
2	3	3	*S.s.*	Muscle	197	28	1.5	*	*	*	0.2	*	*	0.1
2	3	6	*T.l.*	Gills	339	35	7.3	*	2.5	*	0.9	*	0.04	0.1
2	3	6	*T.l.*	Liver	339	35	11.7	3.1	2.8	*	1.5	1.7	0.04	0.6
2	3	6	*T.l.*	Muscle	339	35	2.6	*	*	*	0.3	*	*	0.04
2	3	7	*T.l.*	Gills	400	37	7.6	*	4.7	*	0.9	*	0.1	0.2
2	3	7	*T.l.*	Liver	400	37	14.5	0.8	0.4	*	1.8	0.4	0.01	1.2
2	3	7	*T.l.*	Muscle	400	37	2.2	*	*	*	0.3	*	*	0.1
2	3	8	*T.l.*	Gills	371	34	8.3	*	3.2	*	1.0	*	0.05	0.5
2	3	8	*T.l.*	Muscle	371	34	2.5	*	*	*	0.3	*	*	0.1
2	3	8	*T.l.*	Muscle	371	34	30.0	4.1	3.2	*	3.7	2.2	0.05	*
2	3	9	*T.l.*	Gills	327	32	7.7	*	2.3	*	1.0	*	0.03	0.1
2	3	9	*T.l.*	Liver	327	32	13.1	0.8	0.2	*	1.6	0.4	0.01	0.8
2	3	9	*T.l.*	Muscle	327	32	2.7	*	*	*	0.3	*	*	0.1
2	3	10	*T.l.*	Gills	353	34	7.7	*	6.4	*	1.0	*	0.1	0.1
2	3	10	*T.l.*	Liver	353	34	13.5	1.5	4.0	*	1.7	0.8	0.1	1.0
2	3	10	*T.l.*	Muscle	353	34	1.7	*	*	*	0.2	*	*	0.1
2	3	11	*T.l.*	Gills	291	31	6.6	*	12.3	*	0.8	*	0.2	0.2

2	3	11	*T.l.*	Liver	291	31	14.4	2.9	1.8	*	1.8	1.6	0.03	0.9
2	3	11	*T.l.*	Muscle	291	31	2.0	*	*	*	0.3	*	*	0.2
2	3	12	*T.l.*	Gills	319	34	7.2	*	4.8	*	0.9	*	0.1	0.1
2	3	12	*T.l.*	Liver	319	34	11.3	1.8	1.8	*	1.4	0.9	0.03	0.6
2	3	12	*T.l.*	Muscle	319	34	1.7	*	*	*	0.2	*	*	0.1
2	3	13	*T.l.*	Gills	348	33	8.3	*	5.0	*	1.0	*	0.1	0.4
2	3	13	*T.l.*	Liver	348	33	20.4	1.9	*	*	2.5	1.0	*	3.2
2	3	13	*T.l.*	Muscle	348	33	2.4	*	*	*	0.3	*	*	0.05
2	3	14	*T.l.*	Gills	400	39	8.5	*	5.7	*	1.1	*	0.1	0.2
2	3	14	*T.l.*	Liver	400	39	28.8	1.9	1.6	*	3.6	1.0	0.02	4.5
2	3	14	*T.l.*	Muscle	400	39	1.6	*	*	*	0.2	*	*	0.1
2	3	15	*T.l.*	Gills	306	32	7.5	*	3.4	*	0.9	*	0.05	0.2
2	3	15	*T.l.*	Liver	306	32	10.7	1.2	0.7	*	1.3	0.7	0.01	0.6
2	3	15	*T.l.*	Muscle	306	32	2.3	*	*	*	0.3	*	*	0.1
2	3	16	*T.l.*	Gills	290	38	8.1	*	12.5	*	1.0	*	0.2	0.3
2	3	16	*T.l.*	Liver	290	38	15.6	4.5	1.2	*	1.9	2.4	0.02	1.0
2	3	16	*T.l.*	Muscle	290	38	1.9	*	*	*	0.2	*	*	0.06
2	3	17	*T.l.*	Gills	299	32	7.7	*	3.0	*	1.0	*	0.04	0.2
2	3	17	*T.l.*	Liver	299	32	13.7	1.9	0.8	*	1.7	1.0	0.01	1.3
2	3	17	*T.l.*	Muscle	299	32	1.9	*	*	*	0.2	*	*	0.1
2	4	2	*S.s.*	Gills	213	28	39.7	*	*	*	1.3	*	0.03	0.7
2	4	2	*S.s.*	Liver	213	28	58.0	*	*	*	1.9	*	0.1	11.1
2	4	2	*S.s.*	Muscle	213	28	13.4	*	*	*	0.4	*	*	0.7
2	5	1	*S.s.*	Gills	235	29	55.9	*	*	68.5	5.1	*	0.1	3.4
2	5	1	*S.s.*	Liver	235	29	42.5	*	*	209.9	3.9	*	0.5	10.4
2	5	1	*S.s.*	Muscle	235	29	6.3	*	*	4.2	0.6	*	0.02	0.2
2	5	3	*S.s.*	Gills	172	26	27.2	*	*	6.2	2.5	*	0.1	0.3
2	5	3	*S.s.*	Liver	172	26	46.9	*	*	319.3	4.3	*	0.8	15.8
2	5	3	*S.s.*	Muscle	172	26	7.0	*	*	1.4	0.6	*	*	0.1
3	1	1	*S.s.*	Gills	174	25	58.3	*	1.9	*	1.2	*	0.02	0.6
3	1	1	*S.s.*	Liver	174	25	105.0	*	10.2	*	2.2	13.1	0.1	22.7
3	1	1	*S.s.*	Muscle	174	25	37.6	*	*	*	0.8	*	*	0.1
3	1	3	*S.s.*	Liver	150	25	116.3	*	20.8	*	2.4	5.8	0.3	74.3
3	1	3	*S.s.*	Muscle	150	25	18.5	*	*	*	0.4	*	*	0.05
3	1	4	*S.s.*	Gills	256	31	79.5	*	7.0	*	1.6	*	0.1	0.3
3	1	4	*S.s.*	Liver	256	31	165.6	*	15.0	*	3.4	27.5	0.2	7.7
3	1	4	*S.s.*	Muscle	256	31	16.5	*	*	*	0.3	*	*	0.1
3	1	6	*S.s.*	Gills	237	29	50.5	*	2.1	*	1.0	*	0.03	1.0
3	1	6	*S.s.*	Muscle	237	29	18.4	*	0.7	*	0.4	*	0.01	0.1
3	1	7	*S.s.*	Gills	237	29	53.7	*	3.2	*	1.1	*	0.04	0.4
3	1	7	*S.s.*	Liver	237	29	117.2	*	28.0	*	2.4	20.2	0.3	34.2
3	1	7	*S.s.*	Muscle	237	29	25.8	*	0.7	*	0.5	*	0.01	0.1
3	1	8	*S.p.*	Gills	345	26	85.8	*	4.2	*	1.8	*	0.1	0.2
3	1	8	*S.p.*	Gills	345	26	68.6	*	4.2	*	1.4	*	0.1	0.4
3	1	8	*S.p.*	Liver	345	26	187.3	*	5.5	*	3.8	30.1	0.1	4.7

3	1	8	*S.p.*	Muscle	345	26	38.4	*	*	*	0.8	*	*	0.1
3	1	9	*T.l.*	Gills	261	29	1343	*	*	*	27.5	*	*	0.2
3	1	9	*T.l.*	Liver	261	29	586.2	*	*	*	12.0	1.1	*	0.6
3	1	10	*S.p.*	Gills	206	20	53.8	*	0.8	*	1.1	*	0.01	0.3
3	1	10	*S.p.*	Liver	206	20	79.6	*	*	*	1.6	2.3	*	0.4
3	1	10	*S.p.*	Muscle	206	20	16.3	*	3.2	*	0.3	*	0.04	0.04
3	1	11	*S.s.*	Gills	348	35	75.9	*	3.1	*	1.6	*	0.04	0.3
3	1	11	*S.s.*	Liver	348	35	143.3	*	9.6	*	2.9	5.6	0.1	23.4
3	1	11	*S.s.*	Muscle	348	35	19.0	*	*	*	0.4	*	*	0.1
3	1	12	*S.p.*	Gills	324	25	57.4	*	1.1	*	1.2	*	0.01	0.2
3	1	12	*S.p.*	Liver	324	25	100.6	*	*	*	2.1	2.9	*	0.5
3	1	12	*S.p.*	Muscle	324	25	22.1	*	*	*	0.5	*	*	0.1
3	1	12	*S.p.*	Muscle	324	25	42.1	*	*	*	0.9	*	*	0.2
3	1	13	*S.p.*	Gills	>440	35	69.3	*	*	*	1.4	*	*	0.1
3	1	13	*S.p.*	Muscle	>440	35	34.0	*	11.4	*	0.7	*	0.1	0.1
3	1	13	*S.p.*	Muscle	>440	35	37.4	*	*	*	0.8	*	*	0.1
3	2	7	*S.p.*	Gills	>440	31	38.5	*	0.8	*	1.1	*	0.01	0.2
3	2	7	*S.p.*	Liver	>440	31	78.4	*	*	*	2.2	0.9	*	0.4
3	2	7	*S.p.*	Muscle	>440	31	18.2	*	*	*	0.5	*	*	0.1
3	2	8	*S.p.*	Gills	284	28	42.9	*	6.0	*	1.2	*	0.1	0.1
3	2	8	*S.p.*	Liver	284	28	314.7	*	0.2	*	8.9	1.2	0.01	0.6
3	2	8	*S.p.*	Muscle	284	28	21.5	*	*	*	0.6	*	*	0.1
3	2	9	*S.p.*	Gills	293	24	34.1	*	1.1	*	1.0	*	0.02	0.1
3	2	9	*S.p.*	Liver	293	24	126.9	*	*	*	3.6	0.9	*	0.4
3	2	9	*S.p.*	Muscle	293	24	10.7	*	*	*	0.3	*	*	0.01
3	2	10	*S.p.*	Gills	341	28	25.5	*	2.0	*	0.7	0.8	0.03	*
3	2	10	*S.p.*	Liver	341	28	193.6	*	2.0	*	5.5	14.8	0.04	1.2
3	3	1	*T.l.*	Gills	175	26	31.2	*	3.8	*	0.9	*	0.04	0.1
3	3	1	*T.l.*	Muscle	175	26	13.0	*	*	*	0.4	*	*	0.1
3	3	3	*S.s.*	Liver	196	26	37.2	*	19.7	*	1.1	2.3	0.2	11.4
3	3	3	*S.s.*	Muscle	196	26	7.4	*	0.9	*	0.2	*	0.01	0.03
3	3	4	*S.s.*	Gills	75	20	32.7	*	22.5	*	1.0	*	0.2	0.1
3	3	4	*S.s.*	Liver	75	20	57.6	*	4.5	*	1.7	*	0.04	5.2
3	3	4	*S.s.*	Muscle	75	20	13.0	*	*	*	0.4	*	*	0.03
3	3	5	*T.l.*	Gills	177	27	*	*	*	*	*	*	*	*
3	3	5	*T.l.*	Liver	177	27	72.9	*	2.8	*	2.2	6.8	0.03	1.0
3	3	5	*T.l.*	Muscle	177	27	10.7	*	1.6	*	0.3	*	0.01	0.02
3	3	6	*S.s.*	Gills	148	26	17.0	*	33.3	*	0.5	*	0.3	0.8
3	3	6	*S.s.*	Liver	148	26	29.7	*	6.7	*	0.9	0.4	0.1	14.6
3	3	6	*S.s.*	Muscle	148	26	6.1	*	*	*	0.2	*	*	0.02
3	3	7	*S.s.*	Gills	217	30	29.4	*	7.9	*	0.9	*	0.1	0.1
3	3	7	*S.s.*	Liver	217	30	63.2	*	1.5	*	1.9	1.4	0.01	2.7
3	3	7	*S.s.*	Muscle	217	30	*	*	*	*	*	*	*	*
3	4	1	*T.l.*	Gills	175	26	57.3	*	*	*	0.9	*	0.1	0.1
3	4	1	*T.l.*	Liver	175	26	115.9	*	*	*	1.8	2.4	0.04	1.5

3	4	1	*T.l.*	Muscle	175	26	10.3	*	*	*	0.2	*	*	0.02
3	4	2	*S.p.*	Gills	133	19	70.3	*	*	*	1.1	*	0.1	0.1
3	4	2	*S.p.*	Liver	133	19	264.6	*	*	*	4.0	3.4	*	0.7
3	4	2	*S.p.*	Muscle	133	19	25.3	*	*	*	0.4	*	*	0.04
3	4	4	*S.p.*	Gills	108	18	98.5	*	*	*	1.5	*	0.02	0.1
3	4	4	*S.p.*	Liver	108	18	240.5	*	*	*	3.7	4.7	*	0.3
3	4	4	*S.p.*	Muscle	108	18	36.7	*	*	*	0.6	*	0.01	0.1
3	4	5	*T.l.*	Gills	135	23	53.9	*	*	*	0.8	*	0.04	0.1
3	4	5	*T.l.*	Liver	135	23	60.6	*	*	*	0.9	1.9	0.02	0.3
3	4	5	*T.l.*	Muscle	135	23	14.4	*	*	*	0.2	*	0.1	0.03
3	4	6	*T.l.*	Gills	135	24	57.0	*	*	*	0.9	*	0.1	0.1
3	4	6	*T.l.*	Liver	135	24	77.0	*	*	*	1.2	5.4	0.1	7.8
3	4	6	*T.l.*	Muscle	135	24	15.3	*	*	*	0.2	*	*	0.03
3	5	1	*T.l.*	Gills	124	23	11.7	*	1.8	4.7	1.4	*	0.03	0.1
3	5	1	*T.l.*	Liver	124	23	20.3	*	2.4	40.1	2.5	2.8	0.04	0.9
3	5	2	*S.s.*	Gills	>440	36	9.3	*	3.2	6.8	1.1	*	0.1	0.2
3	5	2	*S.s.*	Liver	>440	36	10.9	*	*	153.4	1.3	0.2	*	3.5
3	5	2	*S.s.*	Muscle	>440	36	3.4	*	*	1.3	0.4	*	*	0.03
3	5	3	*S.s.*	Gills	427	35	10.5	*	23.6	11.5	1.3	*	0.4	0.3
3	5	3	*S.s.*	Liver	427	35	19.5	*	0.4	48.0	2.4	0.4	0.01	1.1
3	5	3	*S.s.*	Muscle	427	35	4.3	*	*	2.1	0.5	*	*	0.05
3	5	4	*S.s.*	Gills	137	28	10.7	*	19.5	7.5	1.3	*	0.4	0.2
3	5	4	*S.s.*	Muscle	137	28	3.1	*	*	1.9	0.4	*	*	0.04
4	1	1	*S.s.*	Gills	193	26	52.1	*	0.4	2.0	1.3	*	0.01	0.2
4	1	1	*S.s.*	Liver	193	26	44.6	*	2.4	47.0	1.1	1.2	0.03	4.8
4	1	1	*S.s.*	Muscle	193	26	20.2	*	*	0.4	0.5	*	*	0.04
4	1	2	*S.s.*	Gills	193	26	62.3	*	2.2	1.1	1.6	*	0.03	0.1
4	1	2	*S.s.*	Liver	193	26	76.5	*	7.2	182.1	1.9	4.1	0.1	18.4
4	1	2	*S.s.*	Muscle	193	26	19.4	*	*	0.5	0.5	*	*	0.05
4	1	3	*S.s.*	Gills	130	24	70.7	*	5.0	1.2	1.8	*	0.1	0.1
4	1	3	*S.s.*	Gills	130	24	60.1	*	*	2.0	1.5	*	*	0.2
4	1	3	*S.s.*	Liver	130	24	113.5	*	12.3	243.6	2.9	4.3	0.1	24.7
4	1	3	*S.s.*	Muscle	130	24	18.4	*	*	0.3	0.5	*	*	0.03
4	1	4	*S.s.*	Gills	122	25	55.2	*	4.4	2.3	1.4	*	0.1	0.2
4	1	4	*S.s.*	Liver	122	25	79.7	*	23.8	81.1	2.0	7.1	0.3	8.2
4	1	4	*S.s.*	Muscle	122	25	15.8	*	*	0.5	0.4	*	*	0.05
4	1	5	*S.s.*	Gills	166	26	43.7	*	4.6	2.7	1.1	*	0.1	0.3
4	1	5	*S.s.*	Liver	166	26	100.1	*	16.0	192.0	2.5	4.4	0.2	19.4
4	1	5	*S.s.*	Muscle	166	26	18.7	*	*.	0.3	0.5	*	*	0.03
4	1	6	*S.s.*	Gills	117	23	6.0	*	3.3	0.3	0.2	*	0.04	0.03
4	1	6	*S.s.*	Liver	117	23	148.1	*	10.8	77.2	3.7	6.0	0.1	7.8
4	1	6	*S.s.*	Muscle	117	23	13.7	*	*	0.3	0.3	*	*	0.03
4	1	7	*S.s.*	Gills	82	20	53.3	*	1.6	1.4	1.3	*	0.02	0.1
4	1	7	*S.s.*	Liver	82	20	17.8	*	1.2	14.5	0.4	0.3	0.01	1.5
4	1	7	*S.s.*	Muscle	82	20	22.2	*	*	0.5	0.6	*	*	0.05

4	1	9	*S.p.*	Gills	149	20	55.1	*	0.8	1.5	1.4	*	0.01	0.1
4	1	9	*S.p.*	Liver	149	20	351.6	*	*	15.8	8.9	2.5	*	1.6
4	1	9	*S.p.*	Muscle	149	20	19.2	*	*	0.4	0.5	*	*	0.04
4	1	10	*D.s.*	Gills	149	20	114.7	*	81.3	1.6	2.9	*	0.9	0.2
4	1	10	*D.s.*	Liver	149	20	145.5	*	23.0	10.2	3.7	13.3	0.3	1.0
4	1	10	*D.s.*	Muscle	149	20	33.6	*	3.8	0.7	0.8	*	0.04	0.1
4	2	2	*D.s.*	Gills	60	36	81.5	*	*	*	2.3	*	0.2	0.2
4	2	2	*D.s.*	Liver	60	36	159.6	*	*	*	4.5	10.3	0.1	1.3
4	2	2	*D.s.*	Muscle	60	36	23.5	*	*	*	0.7	*	*	0.02
4	2	3	*D.s.*	Gills	153	21	69.3	*	*	*	2.0	*	0.1	0.1
4	2	3	*D.s.*	Liver	153	21	72.7	*	*	*	2.1	1.3	0.03	0.6
4	2	3	*D.s.*	Muscle	153	21	47.1	*	*	*	1.3	*	*	0.02
4	4	1	*S.s.*	Gills	202	27	115.9	*	1.3	*	1.9	*	0.01	0.2
4	4	1	*S.s.*	Liver	202	27	79.7	*	15.6	*	1.3	1.3	0.1	16.7
4	4	1	*S.s.*	Muscle	202	27	20.4	*	*	*	0.3	*	*	0.03
4	4	2	*S.s.*	Gills	79	20	70.2	*	*	*	1.2	*	*	0.2
4	4	2	*S.s.*	Liver	79	20	16.7	*	2.6	*	0.3	*	0.02	1.4
4	4	4	*T.l.*	Gills	113	22	81.4	*	2.1	*	1.3	*	0.01	0.2
4	4	4	*T.l.*	Liver	113	22	156.5	*	8.3	*	2.6	2.1	0.1	1.3
4	4	4	*T.l.*	Muscle	113	22	27.7	*	*	*	0.5	*	*	0.04
4	4	9	*S.p.*	Gills	118	17	91.8	*	14.1	*	1.5	*	0.1	0.1
4	4	9	*S.p.*	Liver	118	17	195.7	*	*	*	3.2	4.7	*	1.7
4	4	9	*S.p.*	Muscle	118	17	32.6	*	2.6	*	0.5	*	0.02	0.03
4	4	10	*S.p.*	Gills	88	16	134.6	*	*	*	2.2	*	*	0.1
4	4	10	*S.p.*	Muscle	88	16	24.4	*	*	*	0.4	*	*	0.1
4	4	11	*S.p.*	Gills	101	17	111.6	*	1.1	*	1.8	*	0.01	0.1
4	4	11	*S.p.*	Liver	101	17	262.6	*	*	*	4.3	6.7	*	0.9
4	4	11	*S.p.*	Muscle	101	17	24.5	*	*	*	0.4	*	*	0.04

[a] *S.s.*: *Solea senegalensis*, *S.p.*: *Scorpaena porcus*,*T.l.*: *Trigloporus lastoviza*, *D.s.*: *Diplodus sargus sargus*

* Could not be calculated (Either the metal concentration in the fish or in the sediment was <LD)
